# Comprehensive 3D epigenomic maps define limbal stem/progenitor cell function and identity

**DOI:** 10.1038/s41467-022-28966-6

**Published:** 2022-03-11

**Authors:** Mingsen Li, Huaxing Huang, Bofeng Wang, Shaoshuai Jiang, Huizhen Guo, Liqiong Zhu, Siqi Wu, Jiafeng Liu, Li Wang, Xihong Lan, Wang Zhang, Jin Zhu, Fuxi Li, Jieying Tan, Zhen Mao, Chunqiao Liu, Jianping Ji, Junjun Ding, Kang Zhang, Jin Yuan, Yizhi Liu, Hong Ouyang

**Affiliations:** 1 State Key Laboratory of Ophthalmology, Zhongshan Ophthalmic Center, Sun Yat-sen University, Guangzhou, 510060 China; 2 Center for Stem Cell Biology and Tissue Engineering, Key Laboratory for Stem Cells and Tissue Engineering, Ministry of Education, Zhongshan School of Medicine, Sun Yat-Sen University, Guangzhou, 510080 China; 3 Center for Biomedicine and Innovations, Faculty of Medicine, Macau University of Science and Technology, Macao, China

**Keywords:** Adult stem cells, Chromatin

## Abstract

The insights into how genome topology couples with epigenetic states to govern the function and identity of the corneal epithelium are poorly understood. Here, we generate a high-resolution Hi-C interaction map of human limbal stem/progenitor cells (LSCs) and show that chromatin multi-hierarchical organisation is coupled to gene expression. By integrating Hi-C, epigenome and transcriptome data, we characterize the comprehensive 3D epigenomic landscapes of LSCs. We find that super-silencers mediate gene repression associated with corneal development, differentiation and disease via chromatin looping and/or proximity. Super-enhancer (SE) interaction analysis identified a set of SE interactive hubs that contribute to LSC-specific gene activation. These active and inactive element-anchored loop networks occur within the cohesin-occupied CTCF-CTCF loops. We further reveal a coordinated regulatory network of core transcription factors based on SE-promoter interactions. Our results provide detailed insights into the genome organization principle for epigenetic regulation of gene expression in stratified epithelia.

## Abstract (web summary)

Genome topology provides a structural basis for epigenome-mediated transcriptional regulation in eukaryotes. Here the authors characterized the 3D genome of stratified squamous epithelia. They generated a Hi-C map of human limbal stem/progenitor cells (LSCs) and integrated these data with epigenomics, transcription factor binding maps, and transcriptome data.

## Introduction

Understanding the relationship between three-dimensional (3D) epigenomic architecture and function is critical for unlocking the underlying regulatory circuits of gene expression. Stratified squamous corneal epithelium maintains integrity and homeostasis through the limbal stem/progenitor cells (LSCs) residing in the basal layer of the limbus^1,2^. Adult stem cells undergo self-renewal and differentiation throughout life, which is governed by lineage-restricted transcription factor (TF) networks and epigenetic landscapes^3,4^. The master regulator p63 is essential for self-renewal of stratified epithelial stem cells and initiation of the stratification program^5,6^. The non-keratinized fate of the corneal epithelium is important for ocular surface homeostasis and visual clarity. The corneal epithelium often switches into keratinized epidermal-like epithelium under pathological conditions, such as infection, injury, keratohelcosis, alkaline burn, and squamous metaplasia^7–9^. Our previous study depicted the active and repressive histone modification profiles of LSCs and revealed the epigenetic regulatory mechanism of RUNX1, PAX6, and SMAD3 (RPS) in maintaining the non-keratinizing fate of LSCs^9^. However, due to the lack of high-resolution maps of chromatin 3D organization, it remains unclear how genome topology couples with TF-mediated epigenetic structure to determine LSC function and identity.

The chromosome conformation capture (Hi-C) approach has characterized the multi-hierarchical 3D genome structure that is organized by the architectural proteins CTCF (an insulator protein) and cohesin in mammals^10^. The genome is organized into active compartment A and inactive compartment B^10^. The large-scale A/B compartments are further segregated into megabase-sized topologically associating domains (TADs) and DNA loops that typically occur within TADs^11^. TAD boundaries are demarcated by the CTCF/cohesin complex^11^. While TADs remain largely stable across distinct cell types and species^12^, the epigenetic states and cohesin-associated interaction loops within TADs show cellular specifications^13^. The active promoters are flanked by H3K27ac and H3K4me3, whereas enhancers are enriched for H3K27ac, EP300, and/or H3K4me1^14^. In contrast, inactive elements, including repressive and heterochromatic regions, are marked by H3K27me3 and H3K9me2, respectively^15,16^. These active and inactive regulatory regions bound by chromatin regulators influence gene expression via extensive intra-TAD DNA looping in a cell-type-specific manner^17–19^. Accordingly, the comprehensive 3D epigenomic landscape provides a powerful platform for the spatial regulation of gene transcription.

**Fig. 1:**
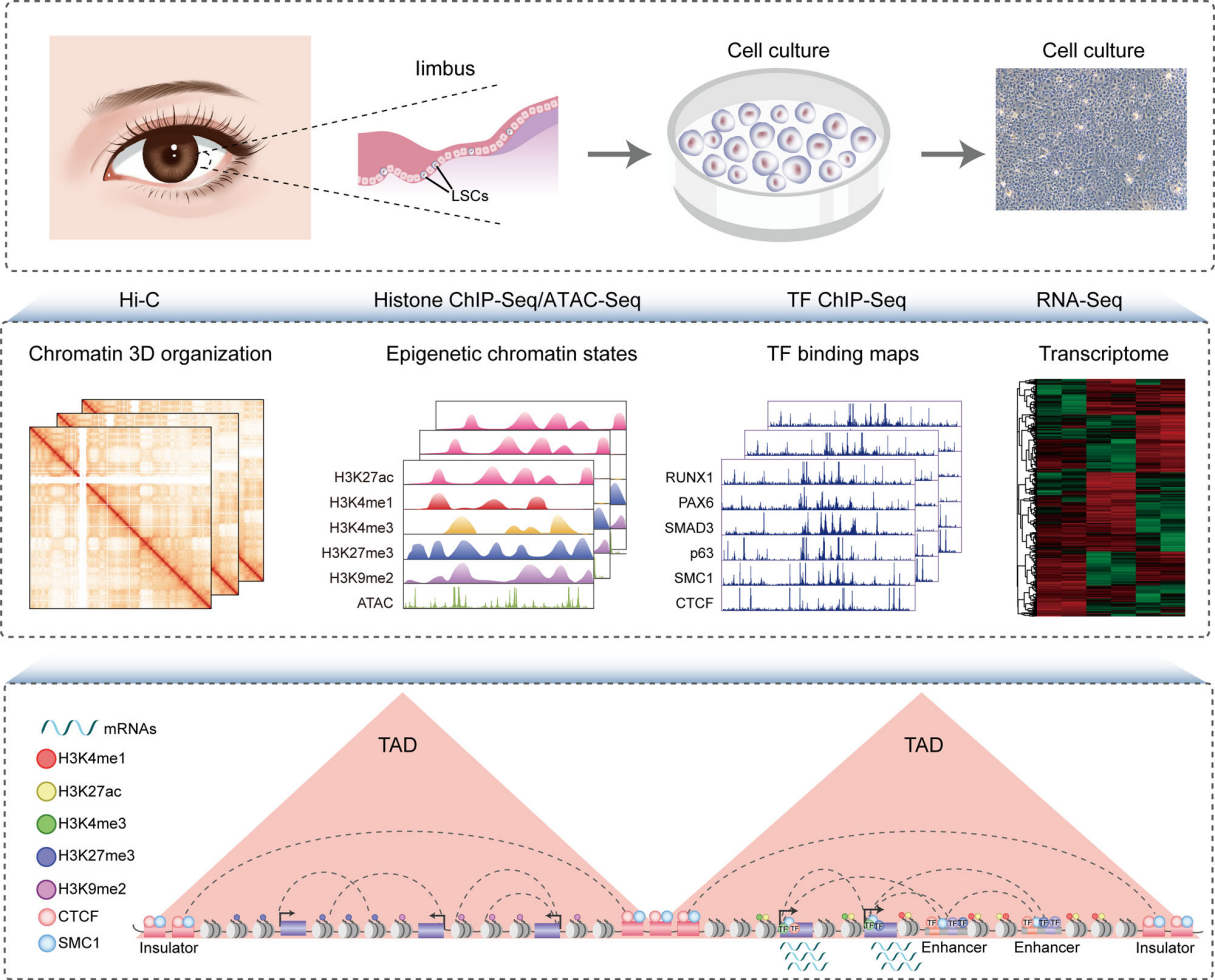
Schematic overview of the study. LSCs were isolated from human limbus biopsies and amplified in vitro. We integrated the data of Hi-C, histone modification, chromatin accessibility, TF binding, and transcriptome to depict a comprehensive 3D regulatory landscape of LSCs.

Here, using Hi-C technology, we characterize the multi-hierarchical genome organization of human LSCs, including chromosomal compartments, TADs, and high-resolution chromatin loops. The combination of chromatin organization, epigenome, TF occupancy, and transcriptome creates comprehensive 3D epigenomic landscapes that contribute to gene activation and repression (Fig. 1). The inactive genome regions with exceptionally high densities of H3K27me3 or H3K9me2 are defined as super-silencers. These super-silencers maintain corneal epithelial identity and repress disease-associated genes through chromatin interactions and/or proximity. We construct super-enhancer (SE) interaction networks that regulate LSC function and identity. The identification of a cohort of intersected SE interaction hubs that contain multiple SE–SE and SE-promoter (SE–P) loops proposes a regulatory pattern of SEs. The active and inactive region-anchored interactions are associated with cohesin and largely occur within the cohesin-occupied CTCF-CTCF loop domains. We further assign the well-known core TFs (p63 and RPS) of LSCs to their target genes via SE–P interactions. Collectively, our high-resolution chromatin interactome characterizes the 3D regulatory landscape of stratified epithelial stem cells.

## Results

### Multiscale 3D genome organization is coupled to transcriptional regulation

**Fig. 2:**
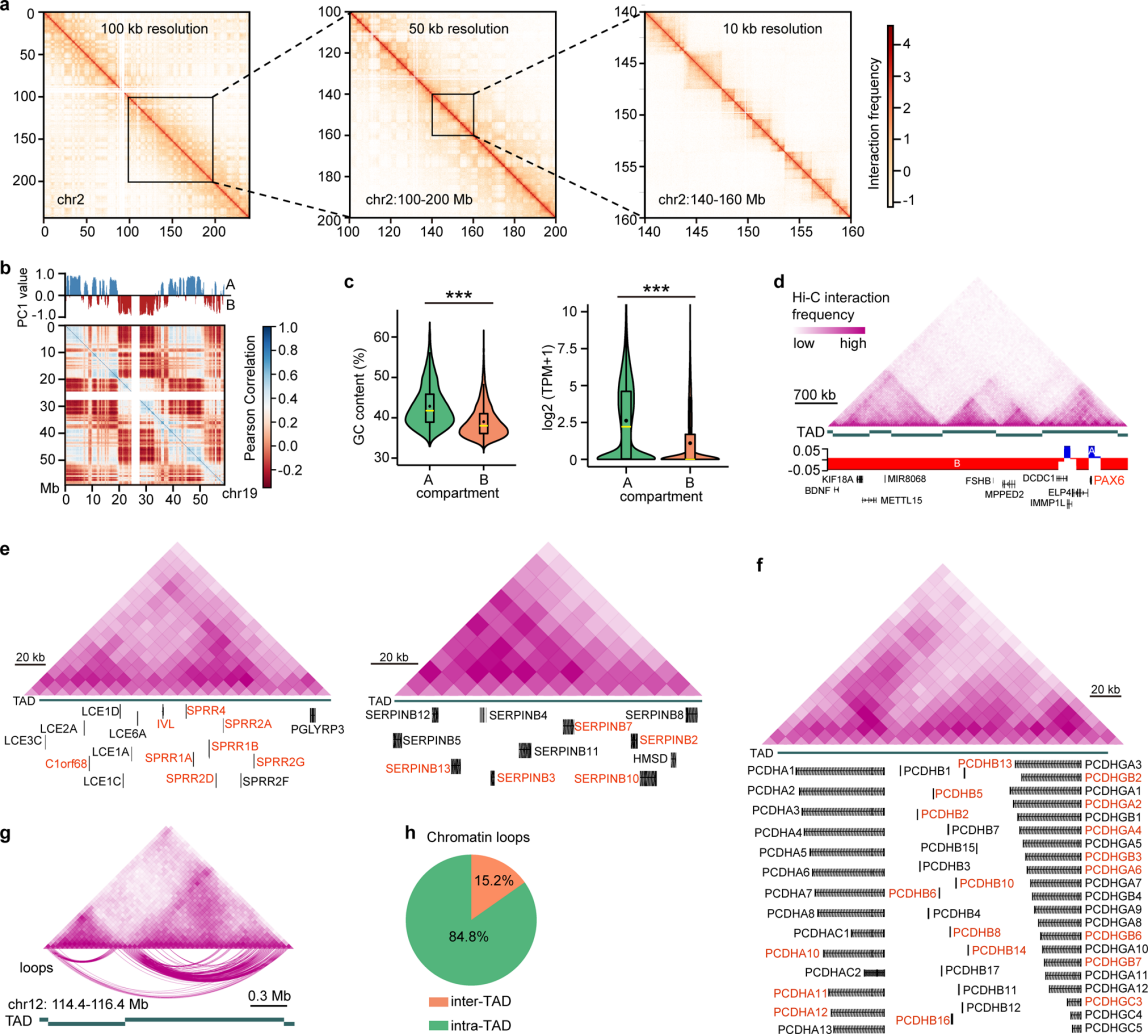
Chromatin multiscale organization of LSCs. **a** Chromatin interaction heatmaps of chromosome 2 at 100-kb, 50-kb, and 10-kb resolution. **b** Chromatin compartments and Pearson correlation heatmap of *cis*-interactions on chromosome 19. Positive first principal component (PC1) values represent compartment A (blue), and negative PC1 values represent compartment B (red). **c** Violin and boxplots showing GC content (left) of A (*n* = 13,613) and B (*n* = 14,149) compartments and gene expression levels in A (*n* = 16,462) and B (*n* = 6,084) compartments. ***p < 0.001 from two-way analysis of variance (ANOVA). The boxplots indicate the 25th percentile (bottom of box), median (horizontal yellow line inside box), mean value (dark spot inside box), and 75th percentile (top of box). Whiskers indicate 1.5 times the interquartile range. TPM: transcripts per kilobase million. **d** Chromatin interaction heatmap, identified TADs, and chromatin compartments at the indicated gene loci. **e**, **f** Chromatin interaction heatmaps of the TADs at the indicated gene loci. The upregulated genes produced by *RUNX1* or *SMAD3* knockdown (**e**) and the genes activated in CECs (**f**) were marked in red. **g** Chromatin interaction heatmap, TADs, and DNA loops at the indicated genomic loci. **h** Percentages of inter-TAD and intra-TAD *cis*-interactions.

To delineate the chromatin higher-order organization principle of LSCs, we conducted in situ Hi-C experiments in two biological replicates with a high degree of correlation (Pearson’s *r* > 0.82). We totally collected ~279 million valid pairwise contacts and generated a genome-wide chromatin interaction profile at a 10-kb resolution by pooling the replicate data (Fig. 2a). Principal component analysis (PCA) of the Hi-C contact matrices identified active A and inactive B compartments (Fig. 2b), with A compartments accounting for 44% of the genome and B compartments for 46%. Compartment A exhibited higher GC content, gene density, and gene expression levels than those of compartment B (Fig. 2c and Supplementary Fig. 1a). We also revealed 3862 large-scale TADs in LSCs with a median size of ~0.68 Mb. While a given TAD tended to have a single compartment type, some TADs showed two different compartment types within each of them, as evidenced by the master regulator *PAX6*, which was organized into compartment A and flanked by compartment B in the TAD (Fig. 2d). We found that the members of gene families related to cellular keratinization, such as LCE, SPRR, and SERPINB families, were arranged in clusters and collectively contained within the same TAD (Fig. 2e). Combined with previously published RNA-Seq data, we found that many members of these families were activated together upon depletion of *RUNX1* or *SMAD3* (Fig. 2e). The clustered cadherin gene superfamily members associated with cell–cell adhesion were also located at a TAD, and multiple members were co-regulated when LSCs were differentiated into corneal epithelial cells (CECs; Fig. 2f). These observations suggested that TADs endowed a high-efficiency co-regulation for genes with functional consistency.

Then, high-confidence chromatin contact loops were identified using the Fit-Hi-C tool^20^, including 73,007 intra-chromosomal loops and 32,560 inter-chromosomal loops (Supplementary Fig. 1b). As expected, most of the *cis*-interactions were contained inside the TADs, but ~15% spanned TAD boundaries (Fig. 2g, h). These loop anchors were distributed at both gene bodies and intergenic regions, with most of them being in non-coding regions (Supplementary Fig. 1c).

### Characterizing the comprehensive 3D epigenomic regulatory landscape of LSCs

**Fig. 3:**
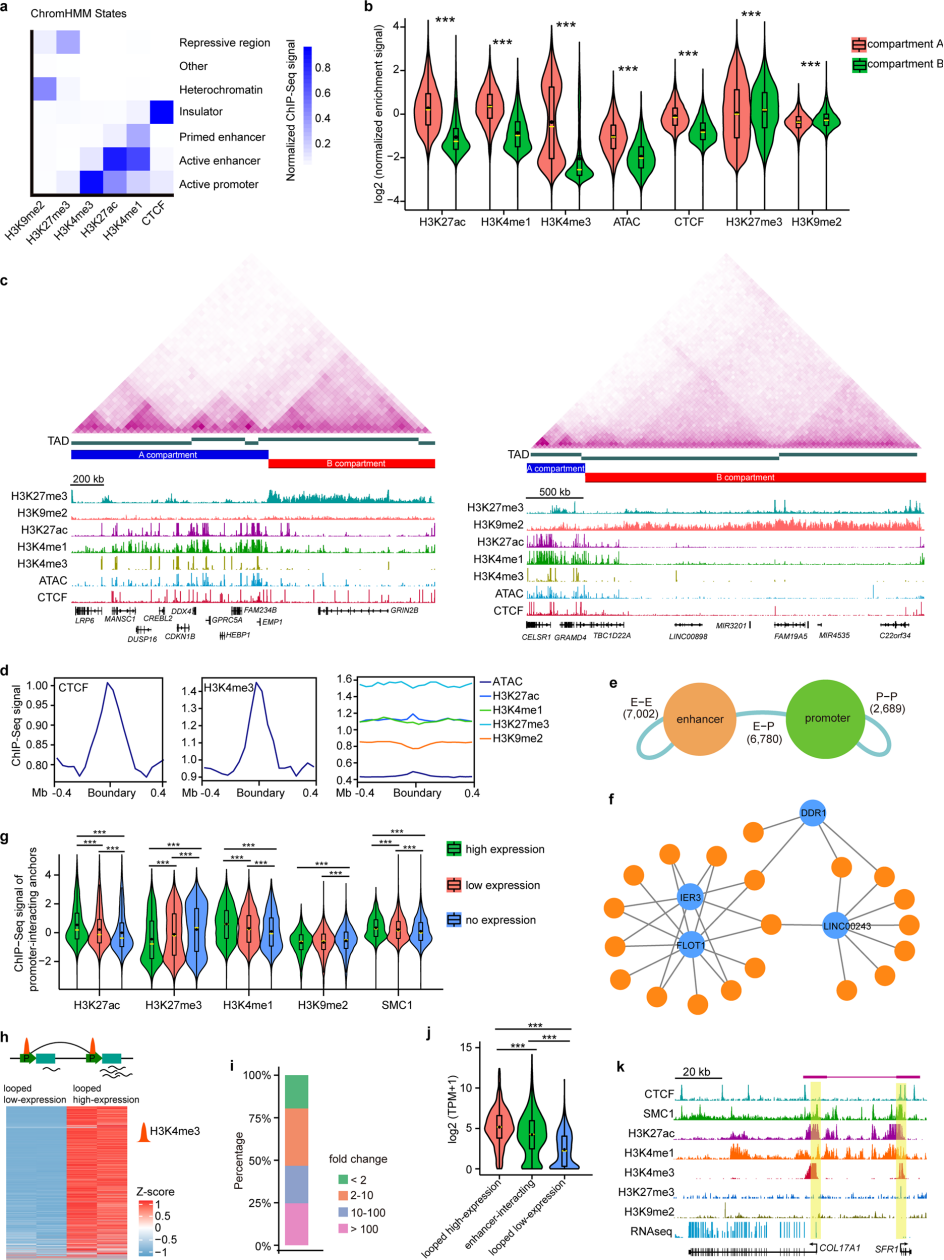
Characterization of a 3D epigenetic regulatory map in LSCs. **a** ChromHMM state annotation using the indicated ChIP-Seq data. **b** Violin and boxplots showing normalized enrichment signal of ATAC-Seq and the indicated ChIP-Seq data at A (*n* = 12,822) and B (*n* = 13,324) compartments. ***p < 0.001 from two-way ANOVA. **c** chromatin interaction heatmaps, identified TADs, chromatin compartments and tracks for enrichment of ATAC-Seq and the indicated ChIP-Seq data at the denoted genomic loci. **d** Metaplots showing the enrichment signal of ATAC-Seq and the indicated ChIP-Seq data at the TAD boundaries. **e** Numbers of E–E, E–P, and P–P loops. **f** Selected E–P interaction network. Orange nodes represent enhancers and blue nodes represent promoters. Edges represent chromatin interactions. **g** Violin and boxplots showing normalized enrichment signal for the indicated ChIP-Seq data at the anchors connected to gene promoters with high (*n* = 8429), low (*n* = 7288) and no (*n* = 7316) expression. High-expression genes: TPM ≥ 10; Low-expression genes: 10 > TPM ≥ 0.1; No expression genes: TPM < 0.1. ***p < 0.001 from two-way ANOVA. **h** Gene expression heatmap of interaction pairs between looped low-expression and looped high-expression promoters. **i** Percentages of the indicated fold changes of looped high-expression promoters/looped low-expression promoters. **j** Violin and boxplots showing gene expression levels of looped high-expression (*n* = 1122), enhancer-interacting (*n* = 2230) and looped low-expression (*n* = 1126) promoters. ***p < 0.001 from two-way ANOVA. **k** A identified chromatin loop and tracks for RNA-Seq and the indicated ChIP-Seq enrichment at the *COL17A1* and *SFR1* loci. All the boxplots indicate the 25th percentile (bottom of box), median (horizontal yellow line inside box), mean value (dark spot inside box), and 75th percentile (top of box). Whiskers indicate 1.5 times the interquartile range.

Our previous work documented the LSC-specific chromatin accessibility landscape and histone modification profiles that mark active (H3K27ac, H3K4me1, and H3K4me3) and repressive (H3K27me3) genomic regulatory elements^9^. In this study, we also performed chromatin immunoprecipitation sequencing (ChIP-Seq) for CTCF and the heterochromatic mark H3K9me2 in LSCs. ChromHMM segmentation annotation based on a combination of these epigenome data identified multiple chromatin states, including active and primed enhancers, active promoters, repressive regions, heterochromatin, and insulators (Fig. 3a). Although both H3K27me3 and H3K9me2 represent the transcriptionally inactive state, their genome-wide occupancies showed distinct patterns, with only 16.7% of the peaks overlapping (Fig. 3a and Supplementary Fig. 2a). As expected, the active histone signatures and accessible regions were preferentially enriched in compartment A, while the repressive H3K27me3 and H3K9me2 were more frequently enriched in compartment B (Fig. 3b). Compartment A also showed a stronger CTCF enrichment signal compared to compartment B (Fig. 3b). The majority of TADs were involved in active histone modifications, but we also observed 244 H3K27me3-marked and 101 H3K9me2-marked TADs with >50% of their regions covered by H3K27me3 and H3K9me2, respectively (Fig. 3c). These repressed and heterochromatic TADs were largely assigned to compartment B and contained ~2000 genes that were expressed at lower levels than those in other TADs (Fig. 3c and Supplementary Fig. 2b). CTCF and H3K4me3 were significantly enriched at TAD boundaries (Fig. 3d). In contrast, other histone marks and ATAC signals were not enriched in the boundary regions.

The non-promoter H3K27ac regions were defined as enhancers. We overlapped the loop anchors with enhancers and promoters, identifying 6,780 enhancer-promoter (E–P), 7002 enhancer-enhancer (E–E), and 2689 promoter-promoter (P–P) loops (Fig. 3e). The enhancer- and promoter-anchored transcription start sites (TSSs) were significantly enriched for active but not repressive histone marks, and enhancer-interacting genes exhibited higher average expression levels than other genes (Supplementary Fig. 2c, d). A lot of enhancers were linked to multiple promoters, and a set of promoters were also targeted by more than one enhancer, establishing an E–P loop regulatory network (Fig. 3f). In particular, the LSC marker genes *KRT15* and *KRT19* were co-regulated by an enhancer network (Supplementary Fig. 2e). Considering the indispensable role of cohesin in establishing E–P loops, we generated a binding map of its subunit SMC1, with 25,711 peaks bound by CTCF (Supplementary Fig. 2f). We found that the gene expression levels were positively correlated with enrichment of H3K27ac, H3K4me1, and SMC1 at the promoter-interacting anchors and negatively correlated with H3K27me3 and H3K9me2 signals in these regions (Fig. 3g).

Interestingly, we focused on the interactions between H3K4me3-marked promoters and found striking gene expression differences between almost all the P–P loop gene pairs (Fig. 3h). The paired anchor promoters were defined as looped low-expression promoters and looped high-expression promoters, respectively (Fig. 3h). Approximately 80% of the anchored promoter pairs showed a > 2-fold change between two groups, and half of them had a fold change > 10 (Fig. 3i). Unexpectedly, the average expression level of looped high-expression promoters was higher than that of active enhancer-interacting promoters, and looped low-expression promoters were expressed at the lowest level (Fig. 3j). Despite H3K4me3 enrichment, a sizeable proportion (84%) of looped low-expression promoters was low- or non-expressed (Supplementary Fig. 2g), suggesting that these promoters may function as putative enhancers. For example, the *SFR1* promoter was enriched for H3K27ac and H3K4me3 signals, but its expression was negligible. This promoter interacted with the active promoter of the highly expressed collagen gene *COL17A1*, a structural component of hemidesmosomes, with two anchors of this P–P loop co-bound by CTCF and SMC1 (Fig. 3k). Interestingly, Gene Ontology (GO) analysis showed that the looped high-expression promoters were associated with coding and non-coding RNA processing, protein synthesis and transport, cell cycle, autophagy, and apoptosis (Supplementary Fig. 2h). This indicated that P–P loops might control cellular general and basic physiological processes.

### CTCF and cohesin-associated chromatin interactions

**Fig. 4:**
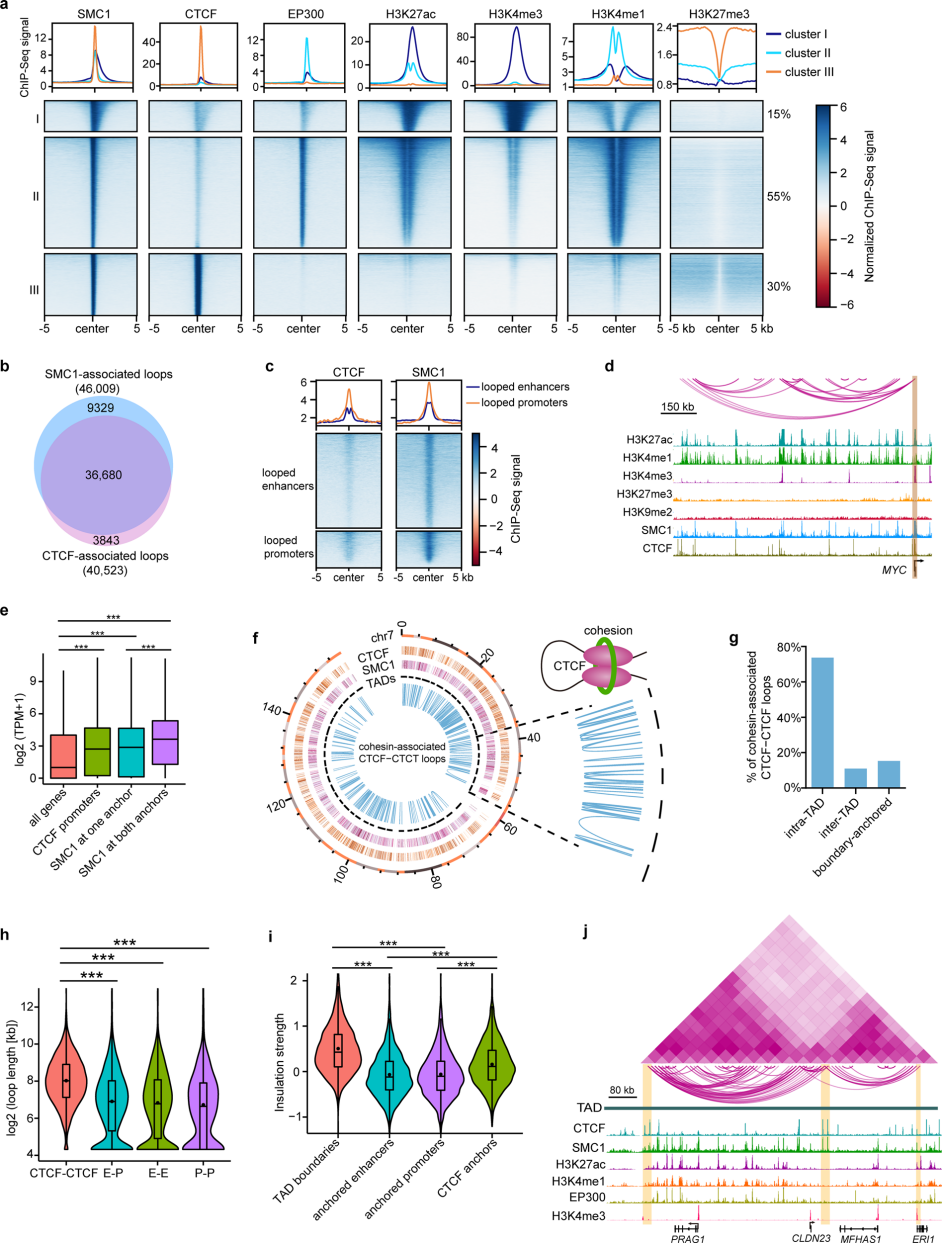
CTCF and cohesin-associated chromatin looping. **a** *K*-means clustering of the indicated ChIP-Seq enrichment at SMC1 sites. **b** Venn diagram showing the overlapping between SMC1- and CTCF-associated chromatin loops. **c** Metaplots and heatmaps showing the enrichment of CTCF and SMC1 at looped enhancers and looped promoters. **d** Identified chromatin loops and tracks for the indicated ChIP-Seq signals at *MYC* locus. **e** Boxplots showing expression levels of all genes (*n* = 25,045), interacting genes with SMC1 at one (*n* = 1265) or both anchors (*n* = 552) and genes with CTCF binding at promoters (*n* = 5877). ***p < 0.001 from two-way ANOVA. Boxplots indicate the 25th percentile (bottom of box), median (horizontal line inside box), and 75th percentile (top of box). **f** Circos plot showing the peaks of CTCF and SMC1, TADs, and cohesion-associated CTCF-CTCF loops on chromosome 4, with zoomed-in regions. **g** Percentages of cohesion-associated CTCF-CTCF loops that were intra-TAD, inter-TAD, and boundary-anchored. **h** Violin and boxplots showing the length of CTCF-CTCF (*n* = 6738), E–P (*n* = 6780), E–E (*n* = 7002) and P–P (*n* = 2689) loops. ***p < 0.001 from two-way ANOVA. **i** Violin and boxplots showing insulation strength of TAD boundaries (*n* = 3876), anchored enhancers (*n* = 4860), anchored promoters (*n* = 4791) and CTCF anchors (*n* = 8731). ***p < 0.001 from two-way ANOVA. **j** Chromatin interaction heatmap, identified loops, and tracks for the indicated ChIP-Seq signals within the indicated TAD. Boxplots (**h**, **i**) indicate the 25th percentile (bottom of box), median (horizontal line inside box), mean value (dark spot inside box), and 75th percentile (top of box). Whiskers indicate 1.5 times the interquartile range.

CTCF and cohesin are the most important architectural proteins that contribute to 3D genome organization^21^. We found that SMC1-occupied sites were grouped into three distinct clusters according to histone modifications and enhancer marker EP300^22^ (Fig. 4a). Cluster I regions (15%) were primarily enriched for H3K27ac and H3K4me3, indicating active promoters. Cluster II sites (55%) showed active enhancer features with the enrichment of H3K27ac, H3K4me1, and EP300. Part of the cluster I and II regions involved relatively weak CTCF binding. Cluster III (30%) sites that were not associated with enhancers and promoters but bound by CTCF were identified as insulators. Despite the depletion of histone modification and EP300, these insulators were flanked by H3K27me3 (Fig. 4a). We identified 46,009 DNA interactions with SMC1 at one or both anchors. There were 40,523 CTCF-associated interactions, most of which involved SMC1 (Fig. 4b). Both the looped enhancers and promoters were significantly enriched for SMC1, and CTCF preferentially occupied some of the looped promoters (Fig. 4c), consistent with their documented roles at these regulatory elements^23,24^. A recent study revealed an interesting finding that CTCF binding at promoters can guide an enhancer to select its target promoter in a cohesin-dependent manner^24^. Indeed, we identified a subset of CTCF-occupied promoters that interacted with the distal enhancers. For example, at the *MYC* locus, CTCF bound to the promoter and multiple E–P and E–E interactions were observed, with SMC1 occupying both anchors (Fig. 4d). Compared to all genes, the genes with CTCF binding at their promoters were expressed at higher levels (Fig. 4e). Likewise, the interacting genes with SMC1 at one or both anchors also showed higher average expression levels than all genes. The anchor genes with SMC1 at both anchors were expressed at higher levels than those with SMC1 at only one anchor (Fig. 4e). These data indicated that SMC1 and CTCF may influence gene transcription by building E–P interactions.

Prior studies have suggested that most CTCF-CTCF loops function as insulated neighborhoods that constrain E–P interactions within the loops in human and mouse embryonic stem cells^25,26^. We also identified cohesin-associated CTCF-CTCF loops that did not involve enhancers and promoters in LSCs, most of which occurred within TADs (Fig. 4f, g). As expected, these insulator-mediated interaction domains were much larger in size than promoter- and enhancer-anchored loops (Fig. 4h), with a median size of 260 kb. Approximately 67% of the E–P loops were contained in these CTCF-CTCF domains. In line with the TAD boundaries, these CTCF-CTCF loop anchors had a larger insulation strength than the anchored enhancers and promoters (Fig. 4i). As exemplified at the *PRAG1* locus, the CTCF interaction anchors divided the TAD into two sub-domains, and plenty of promoter-, enhancer- and insulator-anchored interactions occurred within these two CTCF-CTCF loop domains, with boundary-crossing interactions being confined (Fig. 4j). Our results implied that these cohesin-associated long-range CTCF-CTCF loop structures may define putative insulated neighborhoods.

### Identification and characterization of super-silencer-associated interactions

**Fig. 5:**
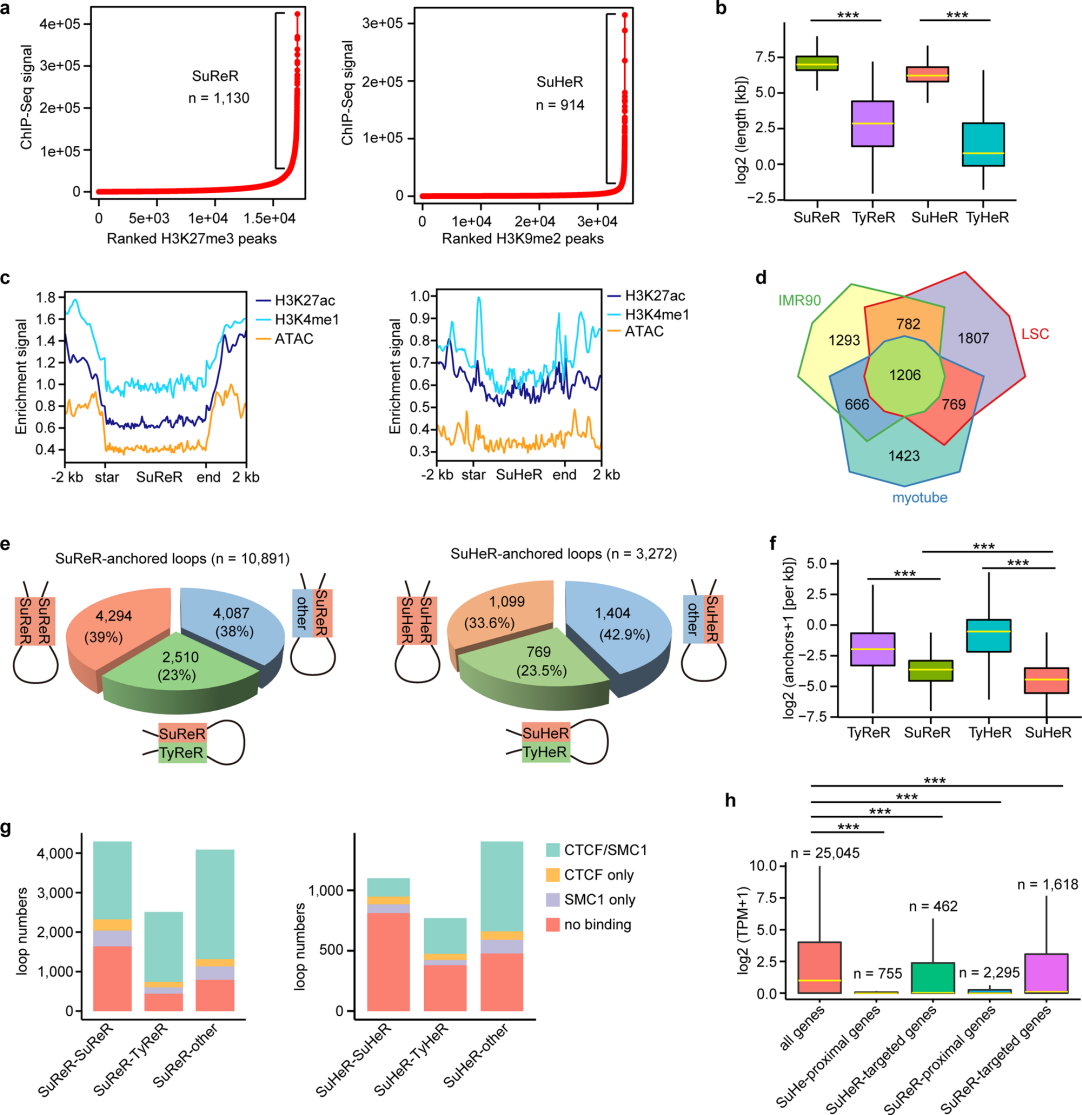
Identification of super-silencers and characterization of super-silencer-associated chromatin contacts. **a** Ranked H3K27me3 and H3K9me2 ChIP-Seq signals. Regions with exceptionally high densities of H3K27me3 and H3K9me2 ChIP-Seq signals are defined as SuReR and SuHeR, respectively. **b** Boxplots showing the sizes of SuReR (*n* = 1130), TyReR (*n* = 15,958), SuHeR (914) and TyHeR (*n* = 33,729). ***p < 0.001 from two-way ANOVA. **c** Metaplots of H3K27ac, H3K4me1, and ATAC enrichment signals at SuReR and SuHeR. **d** Venn diagram of SuReR-associated genes across myotube, IMR-90, and LSCs. **e** Pie charts showing the fractions of the indicated SuReR-anchored and SuHeR-anchored loops. **f** Boxplots showing the interaction densities of SuReR (*n* = 1130), SuHeR (914), TyReR (*n* = 15,958) and TyHeR (33,729). ***p < 0.001 from two-way ANOVA. **g** Fractions of the indicated loops with CTCF/SMC1, SMC1 only and CTCF only at the interaction anchors and without CTCF and SMC1 at the interaction anchors. **h** Boxplots showing the gene expression levels of the indicated groups. ***p < 0.001 from two-way ANOVA. All the boxplots indicate the 25th percentile (bottom of box), median (horizontal yellow line inside box), and 75th percentile (top of box). Whiskers indicate 1.5 times the interquartile range.

Chromatin inactivation is a critical event in lineage commitment, differentiation, and function of stem cells^27,28^. A recent study defined H3K27me3-rich genomic regions as super-silencers that mediate gene repression by chromatin interactions^17^. Analogous to the identification of SEs, we used the ROSE algorithm^29^ to rank the clustered H3K27me3 peaks by ChIP-Seq signals and obtained 1130 H3K27me3-rich regions that were defined as super-repressed regions (SuReR; Fig. 5a). The remaining H3K27me3 peak clusters were defined as typical repressed regions (TyReR). Likewise, we identified 914 H3K9me2-right regions designated as super heterochromatin regions (SuHeR) and a cohort of typical heterochromatin regions (TyHeR; Fig. 5a). Only a small fraction (17%) of overlap between SuReR and SuHeR was observed (Supplementary Fig. 3a). Compared with TyReR and TyHeR, SuReR, and SuHeR were much larger in size (Fig. 5b), with median lengths of 128 and 96 kb and spanning up to 800 and 600 kb, respectively. In this study, we defined SuReR and SuHeR as super-silencers. As expected, these super-silencers and typical silencers were depleted of active histone modifications and chromatin accessibility (Fig. 5c and Supplementary Fig. 3b). Notably, SuReR-associated genes showed cellular specification (Fig. 5d).

We identified 10,891 SuReR-anchored interactions and found that SuReR was more likely to interact with the H3K27me3 regions (Fig. 5e). Similarly, most of the SuHeR-anchored loops encompassed SuHeR-SuHeR and SuHeR-TyHeR interactions (Fig. 5e). Super-silencers showed lower interaction density than typical silencers, and SuReR interacted more frequently than SuHeR (Fig. 5f). Some of the super-silencer- and typical-silencer-associated chromatin contacts exhibited binding of CTCF and SMC1 at one or both anchors (Fig. 5g and Supplementary Fig. 3c), suggesting potential roles of CTCF and SMC1 in interactions between silence elements. We identified 1618 genes that were looped to SuReR and 492 genes that interacted with SuHeR, with 237 genes overlapping between the two groups (Supplementary Fig. 3d). The genes proximal to or distally looped to the super-silencers were primarily inactivated (Fig. 5h), indicating the importance of super-silencer-mediated long-range DNA interactions in gene repression.

### Super-silencers repress genes related to corneal development, differentiation, and disease via chromatin interactions and/or proximity

**Fig. 6:**
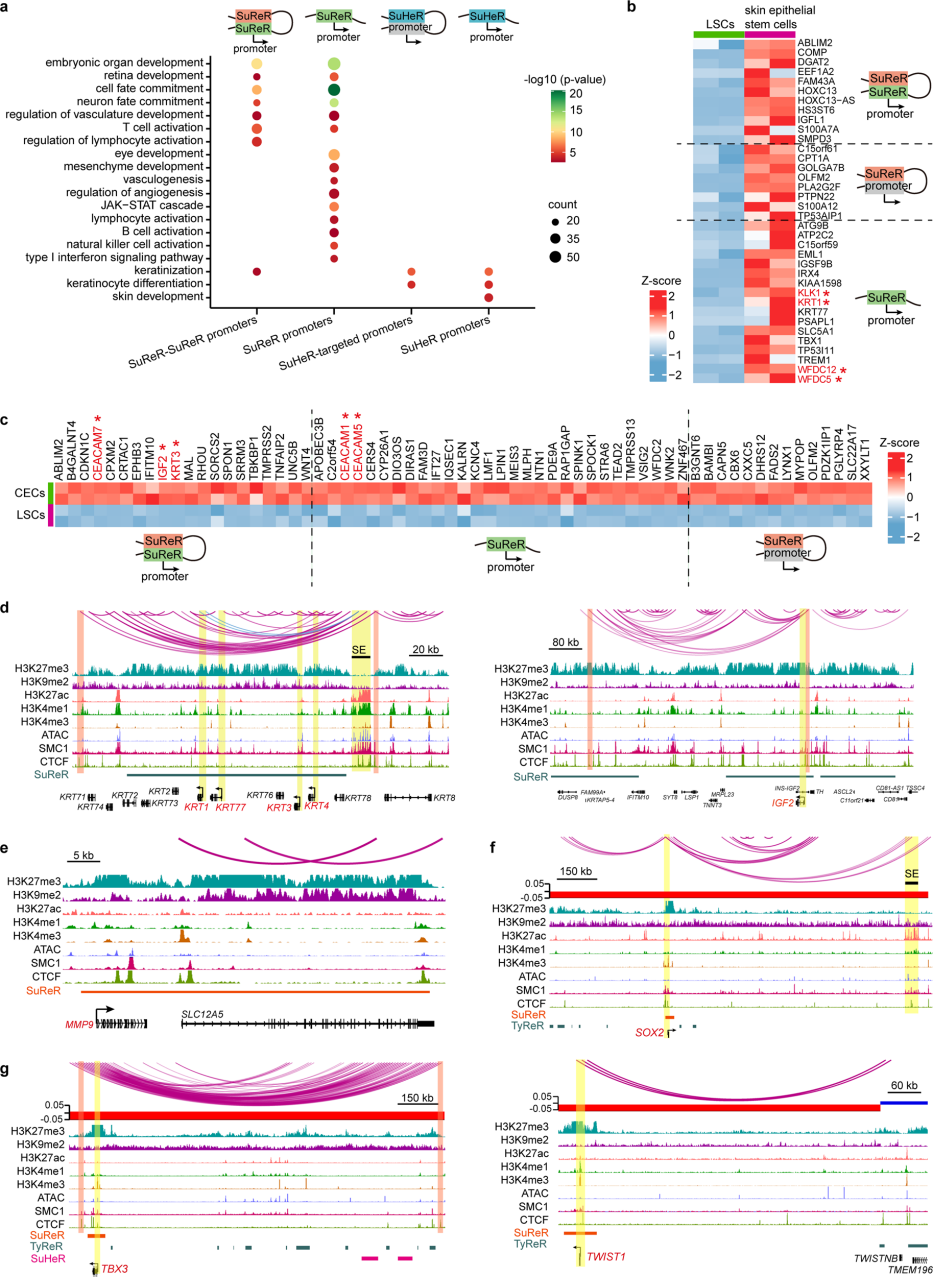
Super-silencers maintain LSC identity and repress disease genes via proximity and chromatin interactions. **a** GO biological process analysis of the indicated groups. **b, c** Heatmaps of the differentially expressed genes between LSCs and skin epithelial stem/progenitor cells (**b**) and between LSCs and CECs (**c**) for the indicated groups. **d**–**g** Identified chromatin loops and tracks for the indicated ChIP-Seq signals at the indicated genomic loci.

To determine the function of super-silencers, we classified SuReR-proximal genes according to whether their promoters interacted with SuReR. GO analysis suggested that both groups were enriched for the embryonic development program and neuron fate commitment (Fig. 6a). PAX6 is a master regulator required for corneal epithelium identity and lineage determination^8,30,31^. PAX6 also dominates neurogenesis and neural fate determination in the nervous system^32–34^. Despite the presence of PAX6, neural fate was turned off by proximal and distal-looping SuReR in LSCs. SuReR-proximal genes were found to involve eye and mesenchyme development. In addition, SuReR inhibited a cohort of genes specifically expressed in skin epithelial stem/progenitor cells, including the well-known epidermal genes *KRT1, KLK1*, *WFDC12*, and *WFDC5*, by proximity and/or interacting with SuReR (Fig. 6b). We then compared the gene expression profiles of LSCs and CECs and found that SuReR repressed a subset of CEC-specific genes in the same manner (Fig. 6c), which was exemplified by the loci of *IGF2*, CEC marker *KRT3*, *CEACAM6*, *CEACAM7*, and *BAMBI* (Fig. 6d and Supplementary Fig. 3e, f). In contrast, the promoters of CEC marker *KRT12*^35^ and epidermis marker *KRT10* were inactive and interacted with distal TyReR and TyHeR (Supplementary Fig. 3g). These observations suggested that super-silencers contributed to the maintenance of stem cell fate, identity, and stemness, as well as gene repression related to development programs.

A previous study indicated that H3K27me3-rich regions could function as tumor suppressors in cancer cells^17^. Therefore, we hypothesized that super-silencers might inhibit disease genes in normal cells. Avascularity, immune privilege, and non-keratinization of the cornea are essential for corneal homeostasis maintenance and clear vision. However, a wide variety of insults, such as infection, injury, squamous metaplasia, keratohelcosis, and genetic diseases, can lead to corneal neovascularization, inflammation, conjunctivalization, and epidermal-like keratinization. Indeed, SuReR-regulated genes showed enrichment for various biological processes associated with immune activation, neovascularization, and keratinization (Fig. 6a). In contrast, SuHeR mediated the repression of skin development, keratinization and keratinocyte differentiation via proximity and chromatin looping (Fig. 6a). For example, *KRT4*, which is activated in conjunctivialized lesions, and keratinization genes *KRT1* and *KRT77* were covered by a large-scale SuReR domain, wherein these promoters formed internal interactions (Fig. 6d). SuReR also overlapped with *MMP9* (Fig. 6e), a widely recognized marker for dry eye syndrome and can disrupt the corneal epithelial barrier and promote corneal neovascularization^36,37^. The corneal epithelium can develop squamous cell carcinoma lesions under pathological conditions^38,39^. We found that the oncogenic TFs *SOX2*^40,41^, *TBX3*^42^, *TWIST1*^41,43^, *DLX5*^44^, and *DLX6*^45^, which are expressed in squamous carcinoma and can promote tumor progression, were located within proximal SuReR domains and interacted with distal TyReR or SuHeR in LSCs (Fig. 6f, g and Supplementary Fig. 3f, h). Interestingly, the promoters of these oncogenic TFs were in a bivalent state primed for expression characterized by H3K4me3/H3K27me3 positive and H3K27ac negative (Fig. 6f, g and Supplementary Fig. 3h). Remarkably, *KRT1* and *SOX2* also interacted with distal SEs, forming active-inactive loops (Fig. 6d, f). Although these disease genes were silenced in LSCs, the bivalent promoters and active-inactive pairwise contacts primed them for activation during homeostasis, which provided a 3D epigenetic basis for disease occurrence. We further found that a subset of super-silencer-associated genes in LSCs, including the above oncogenic TFs, exhibited activated states characterized by significant H3K27ac enrichment in squamous carcinoma (Supplementary Fig. 3i–k), suggesting that super-silencer might maintain tissue homeostasis via repressing disease-associated genes. SuReR showed a higher H3K27ac density than TyReR in squamous carcinoma, but no significant differences of H3K27ac enrichment were observed between SuHeR and TyHeR (Supplementary Fig. 3l), indicating that SuReR may be more likely to be activated than TyReR in tumors. We also found that CTCF and SMC1 occupied some of these inaccessible interaction anchors. In agreement with the E–P loops, these super-silencer-mediated interactions occurred mainly within the CTCF-CTCF loops (Fig. 6d–g and Supplementary Fig. 3e, h). These results indicated that CTCF and cohesin also involved inactive region-mediated chromatin interactions.

Taken together, our data highlighted that proximal super-silencers and super-silencer-mediated chromatin interactions contribute to gene repression associated with corneal epithelial development, differentiation, and disease.

### Spatial clustering of SEs forms 3D SE interactive hubs

**Fig. 7:**
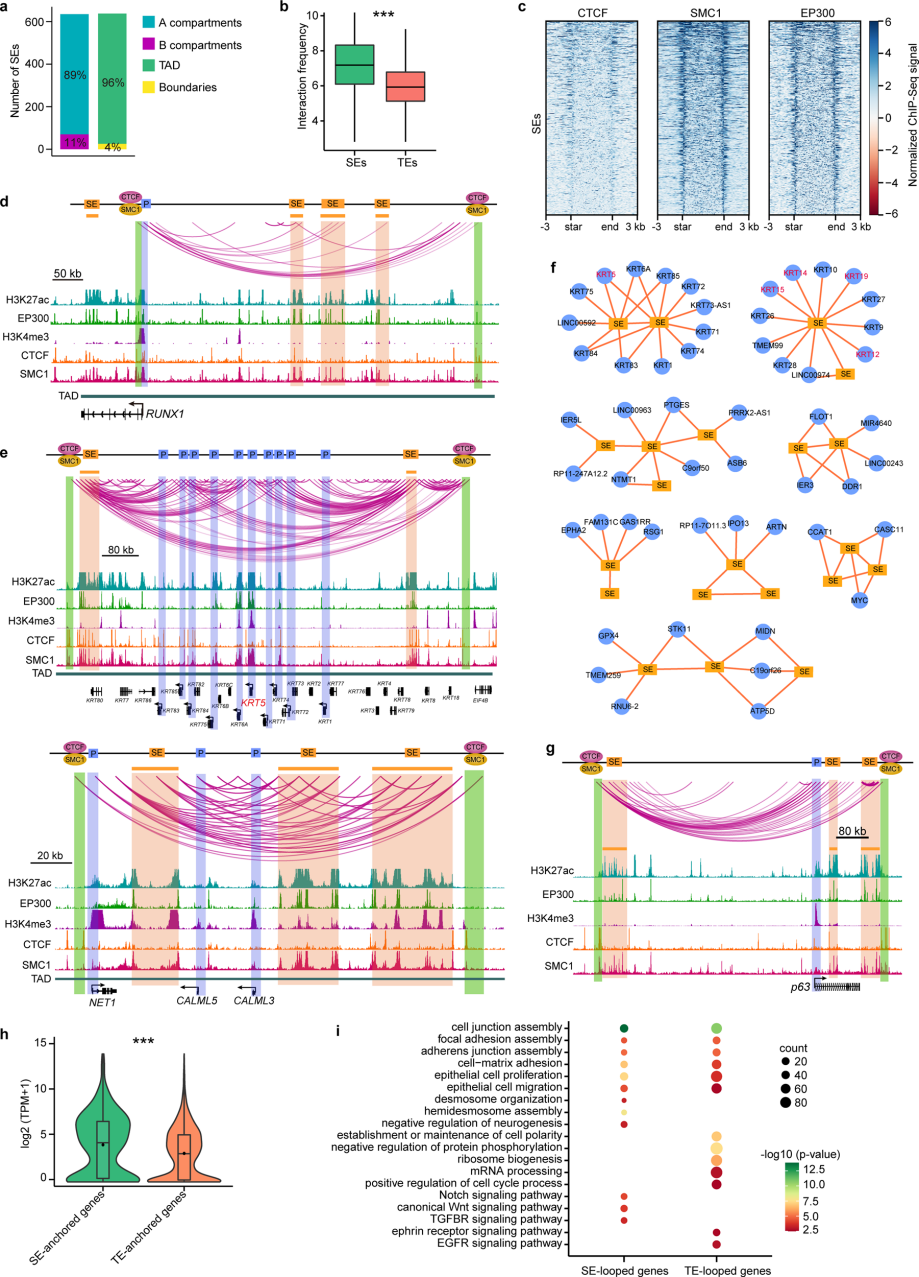
SE-anchored chromatin interaction networks. **a** Numbers of the SEs that are located at A and B compartments and located in TADs and TAD boundaries. **b** Boxplots showing the interaction frequencies of SEs (*n* = 535) and TEs (*n* = 8167). Boxes indicate the 25th percentile, median and 75th percentile. Whiskers indicate 1.5 times the interquartile range. ***p < 0.001 from two-way ANOVA. **c** Heatmaps showing the ChIP-Seq signals of CTCF, SMC1, and EP300 at SEs. **d**, **e** Identified chromatin loops and tracks for the indicated ChIP-Seq signals at the indicated loci. **f** Selected SE-anchored interaction hubs. Edges represent chromatin interactions. **g** Identified chromatin loops and tracks for the indicated ChIP-Seq signals around *p63* locus. **h** Violin and boxplots showing the expression levels of SE-anchored (*n* = 589) and TE-anchored (*n* = 3855) genes. ***p < 0.001 from two-way ANOVA. Boxplots indicate the 25th percentile (bottom of box), median (horizontal line inside box), mean value (dark spot inside box), and 75th percentile (top of box). Whiskers indicate 1.5 times the interquartile range. **i** GO biological process analysis of SE-anchored and TE-anchored genes. *P*-values were calculated by hypergeometric distribution test.

Given the importance of SEs in cell identity^46,47^, we next investigated how the chromatin 3D structure endows the regulatory function of SEs. We found that the vast majority of SEs were assigned to compartment A and inside the TADs (Fig. 7a). Compared with typical enhancers (TEs), SEs formed chromatin looping much more frequently (Fig. 7b). SMC1 and EP300 were significantly enriched in SEs, but few SEs exhibited CTCF binding (Fig. 7c). We identified a set of SE–P loops with some SEs targeting multiple genes and some genes anchored by more than one SE. RUNX1 is required for the maintenance of fate and identity of the corneal epithelium^9^. As shown at the *RUNX1* locus-associated TAD with four SEs, *RUNX1* interacted with three distal upstream SEs but not the closest one within the gene body (Fig. 7d and Supplementary Fig. 4a). The *RUNX1*-SE loops were flanked by two interacting CTCF insulators co-bound by SMC1. CTCF insulators may restrict the interaction between *RUNX1* and the SE in the gene body. At the keratin gene cluster loci, two SEs frequently interacted with many keratin genes, including the stratified epithelial marker *KRT5* (Fig. 7e and Supplementary Fig. 4a). Multiple interactions between these two SEs were also observed. Similarly, three SEs frequently interacted with each other and targeted the promoters of *NET1*, *CALML3*, and *CALML5* in the TAD, forming an SE interaction hub (Fig. 7e). In view of this discovery, we built SE-mediated interaction networks and defined a set of intersected SE interaction hubs consisting of spatially clustered SEs and at least one promoter and involving multiple SE–SE and SE–P loops (Fig. 7f). Notably, in these SE interaction hubs, while some SEs did not directly loop to promoters, they were indirectly associated with promoters by connecting to the promoter-interacting SEs (Fig. 7f). For example, two SEs located downstream of *p63* were spatially close to the *p63* promoter by looping to the upstream SE that interacted with the promoter (Fig. 7g and Supplementary Fig. 4a). CEC-specific *KRT12*, together with LSC markers *KRT14*, *KRT15*, and *KRT19*, were also organized into an SE interaction hub (Fig. 7f). The pre-established SE-*KRT12* loop in LSCs was primed for activating *KRT12* upon differentiation. The SE–P and SE–SE anchors were enriched for SMC1, but only a small fraction showed CTCF binding, and EP300 occupied the interacting SE anchors (Fig. 7d, e, g). Cohesion may contribute to the spatial clustering of SEs, which has been demonstrated in thymocytes^48^. The SE interaction hubs were primarily constrained within the cohesin-occupied CTCF-CTCF loop structures (Fig. 7e, g), which was in line with the SE domains reported previously^26^.

SE-anchored genes showed higher expression levels than TE-anchored genes (Fig. 7h). Both SE- and TE-interacting genes were enriched for the GO terms associated with the pan-epithelial properties: cell junction, focal adhesion, adherens junction, cell-matrix adhesion, proliferation, and migration (Fig. 7i). SEs looped to genes related to the assembly of desmosomes and hemidesmosomes. Notably, LSC-specific SEs repressed neural fate by interacting with negative regulators of neurogenesis (Fig. 7i). In contrast, TE-anchored genes were involved in establishing and maintaining cell polarity and several general biological processes like protein phosphorylation, ribosome biogenesis, and mRNA processing (Fig. 7i). In addition, the genes encoding NOTCH, WNT, and TGFBR signaling pathways that are important to corneal epithelial identity^49,50^ were regulated by SE-mediated chromatin looping. TEs interacted with genes related to the ephrin receptor and EGFR signaling pathways (Fig. 7i). Collectively, we showed that SEs regulated key gene expression through chromatin interaction networks. Genome interaction data can reveal a more comprehensive assignment of SEs to target genes than the proximity principle.

### Core TFs define LSC identity through SE–P interaction networks

**Fig. 8:**
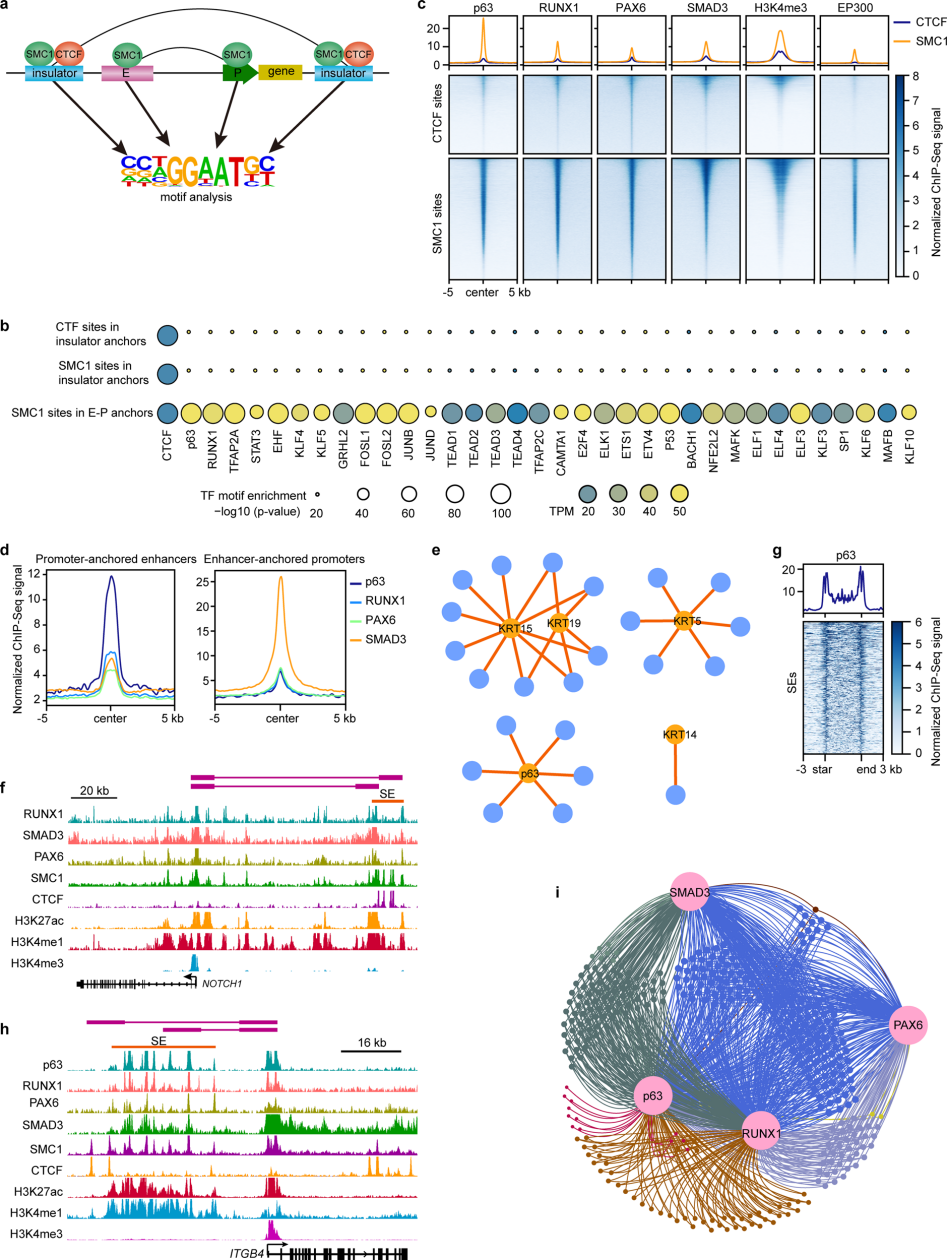
Core TFs regulate LSC function and identity by SE–P interaction networks. **a** Schematic of TF motif enrichment analysis. **b** Identified TF motifs in the indicated groups. **c** Metaplots and heatmaps showing the enrichment of the indicated ChIP-Seq signals at the CTCF and SMC1 sites. **d** Metaplots showing the enrichment of p63 and RPS at promoter-anchored enhancers and enhancer-anchored promoters. **e** Chromatin interactions between p63-bound enhancers and selected genes. Blue nodes represent p63-bound enhancers and orange nodes represent target genes. Edges represent chromatin interactions. **f** Identified chromatin interactions and tracks for the indicated ChIP-Seq signals at *NOTCH1* locus. **g** Metaplot and heatmap showing the enrichment of p63 at SEs. **h** Identified chromatin interactions and tracks for the indicated ChIP-Seq signals at *ITGB4* locus. **i** p63 and RPS mediated an intersected gene regulatory network via SE–P interactions.

The interactions between TFs and *cis*-elements allow us to understand the regulatory circuits for cell-type-specific gene transcription. To determine the TFs involved in chromatin looping, we performed motif analysis for CTCF and SMC1 sites in interacting insulators and SMC1 sites in E–P anchors, respectively (Fig. 8a). The E–P anchors, but not insulators, exhibited significant enrichment of TF motifs for the well-known key regulators p63, RUNX1^9^, TFAP2A^51^, EHF^52^, STAT3^53^, KLF4^54^, KLF5^55^, and AP-1 complex^56^ (FOSL1, FOSL2, JUNB, and JUND). We also identified a set of E–P loop-associated TFs, such as the TEAD family, TFAP2C, ETS family, MAFK, MAFB, KLF6, and KLF10 (Fig. 8b), implying their potential roles in LSCs. As expected, the CTCF binding motif was found in the interacting insulators and E–P anchors (Fig. 8b).

Our previous study generated ChIP-Seq data of SE-associated RPS and highlighted their critical role in LSC identity and fate determination^9^. Due to the strong enrichment (*p*-value = 10^−411^) of the p63 motif in E–P anchors and the master role in stratified epithelia, in this study, we also portrayed the genome-wide binding map of p63 by ChIP-Seq in LSCs. Most p63 peaks were located at introns and intergenic regions, while ~14% were located at the promoters (Supplementary Fig. 4b). Although some overlaps among p63 and RPS peaks were observed, p63 showed a unique binding pattern (Supplementary Fig. 4c), consistent with its distinct functions. The CTCF insulators that were H3K4me3- and EP300-negative did not show p63, RUNX1, and SMAD3 binding, but few CTCF bound active promoters were co-occupied by them (Fig. 8c). However, most CTCF sites showed weak PAX6 binding (Fig. 8c). In contrast, p63 and RPS co-occupied SMC1 sites that were defined as active promoters (H3K4me3-positive) and enhancers (EP300-positive; Fig. 8c). The E–P anchors exhibited a striking enrichment of p63 and RPS, with p63 preferentially at enhancers and SMAD3 preferentially at promoters (Fig. 8d), suggesting that these TFs function via chromatin looping. Interestingly, we found that SMAD3, but not p63, PAX6, and RUNX1, was enriched in TAD boundaries (Supplementary Fig. 4d). RPS-associated chromatin loops were involved in TGF-beta, WNT, P53, p63, and EGF/EGFR pathways and a set of pathways that were not previously well-investigated in LSCs, such as vitamin D receptor, insulin, gastrin, IL4/IL13, PI3K-Akt, and Hippo signaling pathways (Supplementary Fig. 4e), implying a potential role of these pathways in the corneal epithelium.

We showed that p63 regulated the LSC-specific *KRT19* and general stratified epithelial stem cell genes *KRT14*, *KRT15,* and *KRT5* via long-range chromatin interactions (Fig. 8e). p63 also activated itself via multi-loop interactions. Consistent with the well-known function of p63, p63 binding site-anchored genes showed striking enrichment of GO terms associated with epithelial development, cell migration, cell cycle, and pan-epithelial identity (Supplementary Fig. 4f). We also identified multiple biological pathways that were regulated by p63-mediated interactions, including response to EGF stimulus, TGFBR, PDGFR, and p53 signaling pathways. In addition, p63-mediated chromatin interactions participated in histone modification and phospholipid biosynthesis and metabolism (Supplementary Fig. 4f), implying a putative regulatory role of p63 in these processes. We also found that, although not expressed, a subset of the CEC-specific differentiation genes interacted with H3K27ac-marked enhancers in LSCs, and p63 involved most of these pre-established E–P loops (Supplementary Fig. 4g). RPS and NOTCH1^49^ are required for corneal epithelial identity. We previously showed that RPS physically forms a protein complex that co-occupies SEs^9^. We found that RPS regulated the expression of *NOTCH1* by binding to its promoter and the promoter-interacting SE (Fig. 8f). SEs were also significantly enriched for p63 (Fig. 8g). The active promoters of *ITGA4* and *ITGA6* (two genes encoding structural proteins of hemidesmosomes) were connected to distal SEs that were co-occupied by p63 and RPS (Fig. 8h and Supplementary Fig. 4h). These interacting regulatory elements also exhibited SMC1 binding. By integrating Hi-C interaction data, TF binding maps, and SE category, we found that p63 and RPS formed an intersected regulatory network via SE-P interactions. In this regulatory network, most genes were controlled by at least two TFs (Fig. 8i).

## Discussion

**Fig. 9:**
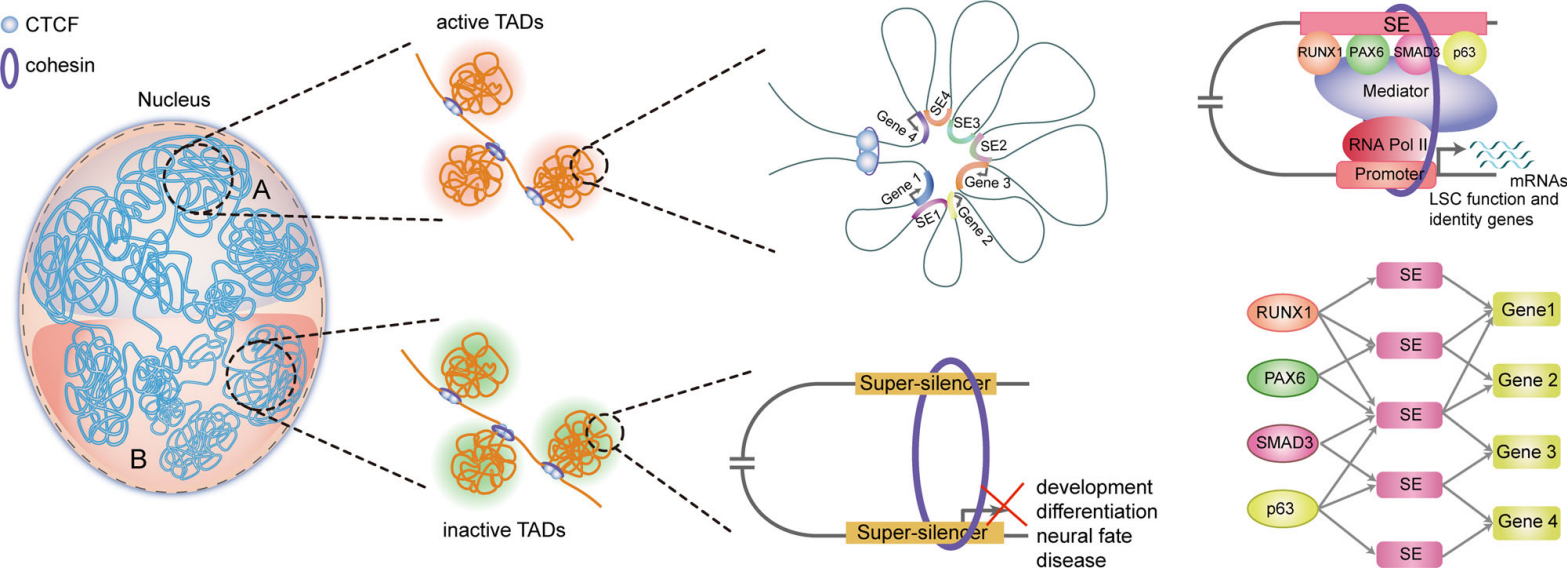
A model of 3D regulatory network organized into the multi-hierarchical genome architectures in LSCs. Genome is organized into compartment A and B that contain active and inactive TADs, respectively. In the active TADs, SEs interact with other SEs and/or target promoters, forming intersected SE 3D interaction hubs that are constrained within cohesion-associated CTCF-CTCF loops. p63 and RPS activate LSC identity genes by binding SEs that interact with target promoters. p63 and RPS establish a coordinated regulatory network through SE-P interactions. In the inactive TADs, super-silencers repress genes associated with development, differentiation, neural fate, and disease via proximity and/or chromatin interactions.

Genome topology provides a proper structural basis for TF- and epigenome-mediated transcriptional regulation in eukaryotes. However, how this process underlies LSC function and identity is poorly understood. In our previous studies, we delineated the histone modification maps associated with promoters, enhancers, and repressors and identified RPS as core TFs required for LSC fate determination^9^. In this study, we first created a high-resolution Hi-C interaction map of human LSCs, providing insights into multi-hierarchical regulation of gene expression. The identification of LSC-specific chromatin higher-order architectures, including A/B compartments, TADs, and DNA loops (Fig. 9), allows for the future investigation into the relationship between structure and function of *cis*-elements. Our high-resolution chromatin loops can be used to precisely annotate enhancers to target genes. We identified and characterized the SE- and super-silencer-mediated chromatin interactions that were organized into active and inactive TADs, respectively (Fig. 9). These active and inactive chromatin interactions were associated with cohesin and were constrained inside the insulated neighborhoods established by the CTCF/cohesin complex. These multi-omic data combination analyses uncovered distinct epigenetic properties of the chromatin 3D structures, which will likely help propose regulatory principles underlying cellular specification in further in-depth studies. Furthermore, we showed p63- and RPS-mediated SE–P interaction networks (Fig. 9). In summary, we provided valuable multi-omic data sources for LSC research and highlighted the gene regulatory network for LSC function and identity at multiple hierarchical levels of DNA interactions. The disorder of chromatin 3D organization is often linked to human diseases^57^. Our chromatin interaction profile provides a theoretical basis for future stem cell-based regenerative therapies.

It has been well-established that gene silence plays a key role in embryonic development and stem cell differentiation^27,28^. A recent study defined H3K27me3-rich genomic regions as super-silencers in cancer cell lines and indicated that cancer-specific super-silencers mediate the repression of tumor suppressor genes via chromatin looping^17^. In our study, we defined H3K27me3-rich and H3K9me2-rich regions as super-silencers in LSCs. Consistent with SEs, super-silencers also showed cellular specification (Fig. 5d). SuReR prevented LSCs committing to neural fate and epidermal lineage and inhibited the differentiation program (Fig. 6a–d), suggesting that super-silencers may play a critical role in lineage determination and stemness maintenance of adult stem cells. Importantly, LSC-specific super-silencers repressed biological processes associated with common corneal pathological alterations like immune activation, neovascularization and keratinization (Fig. 6a). The key oncogenic TFs of squamous cell carcinoma were covered by SuReR and interacted with other silencer elements (Fig. 6e–g and Supplementary Fig. 3h), revealing a regulatory mechanism explaining the prevention of tumorigenesis. However, the bivalent state and/or interaction with SEs of the oncogenic TF promoters may endow epithelial tissues with the potential to transform into tumors. Thus, we highlighted the critical role of super-silencers in tissue homeostasis and disease repression, which would provide theoretical guidance for prevention and treatment of corneal epithelial diseases.

Our data showed that some enhancers, including SEs, can target more than one promoter, and some promoters are connected to one or more distal enhancers (Figs. 3f, 7f). Therefore, chromatin loop structures allow us to identify the precise regulatory elements of target genes and elucidate a more complex enhancer regulatory network. Interestingly, we found that although some SEs did not loop to the promoters in the same TAD, they were indirectly associated with some promoters by interacting with the promoter-anchored SEs (Fig. 7e–g). We hypothesized that these indirect SEs might also activate the expression of genes spatially close to them. The corneal epithelial key and marker genes were organized into these SE interaction hubs, highlighting the importance of this regulatory pattern. Our Hi-C data provided multi-layer regulatory principles for the control of stratified epithelial gene expression.

It is thought that the CTCF/cohesin complex mediates the interactions between insulators and, cohesin is required for establishment and/or maintenance of enhancer-promoter (E–P) loops^11,21^. We showed that SMC1 occupied both regulatory elements and insulators (Fig. 4a). The observation that the genes with SMC1 binding at the E–P anchors were expressed at a higher level indicated an important role of SMC1 in gene transcription. Recent studies have indicated that E–P loops largely occur within insulated neighborhoods^25,26^. Indeed, we found that the E–E, E–P, P–P, and SE–P loops were flanked by two interacting CTCF sites. Similarly, super-silencer-mediated interactions were also contained inside the putative insulated neighborhoods. Despite the major insulator function of CTCF, we also found that CTCF occupied a subset of enhancer-anchored promoters. The genes with CTCF binding at promoters exhibited a relatively higher expression level (Fig. 4e), which can be explained by a recent observation that CTCF binding at promoters can bridge the promoters to distal enhancers^24^.

## Methods

### Ethics statement

This study was conducted in accordance with the criteria set by the Declaration of Helsinki and was approved by the Ethics Committee of Zhongshan Ophthalmic Center of Sun Yat-sen University.

### Cell culture

LSCs were isolated and cultured as previously described^8,9^. Briefly, human limbus biopsies from donors were cut into small pieces and digested with 0.2% collagenase IV (Gibco) and then 0.25% trypsin–EDTA (Gibco). After low-speed centrifugation, precipitates were seeded on the Matrigel-coated polystyrene plates and cultured with LSC medium. The components of LSC medium refer to the previous publication^9^. All human limbus tissues were obtained from four male donors aged from 20 to 46 years with the approval of the Ethics Committee of Zhongshan Ophthalmic Center of Sun Yat-sen University. These donors do not have any ocular surface diseases.

### In situ Hi-C library construction

1 × 10^7^ cells were used for Hi-C. Following cellular cross-linking with 1% formaldehyde and nuclei extraction in hypotonic solution, the genomic DNA was digested with restriction enzyme MboI (NEB) at 37 °C overnight and labeled with biotinylated nucleotides when the cohesive ends were filled. Then, blunt-end proximity ligation was performed with T4 DNA ligase (NEB) at 16 °C for 4 h. After de-crosslinking with proteinase K (Thermo) at 65 °C overnight, ligated DNA was purified by QIAamp DNA Mini Kit (Qiagen) and sheared to ~400 bp. Next, Dynabeads™ MyOne™ Streptavidin C1 (Invitrogen) was used to pull down the biotin-marked ligation junctions. The Hi-C library was generated using NEBNext Ultra™ II DNA Library Prep Kit (NEB) according to the manufacturer’s instructions and then sequenced with paired-end 150 reads using Illumina HiSeq X10.

### Chromosome conformation capture (3 C) assay

Cells were fixed in 1% formaldehyde solution for 15 min and lysed with Hi-C lysis buffer (0.1%SDS; 50 mM HEPES–KOH, pH 7.5; 150 mM NaCl; 1 mM EDTA; 1% Triton X-100; 0.1% Sodium Deoxycholate). Permeable cells were treated with 0.5% SDS at 62 °C for 8 min and 1% Triton X-100 at 37 °C for 15 min. Next, chromatins were digested with MboI restriction enzyme (NEB) overnight at 37 °C and DNA Polymerase I (NEB) was added to incubate for 1 h. The digested DNA fragments were ligated with T4 DNA Ligase (NEB) for 4 h. Decrosslinking was performed with Proteinase K at 55 °C for 1 h. The ligated DNA fragments were purified with ethanol and sodium acetate. qRT-PCR was used to quantify 3C enrichment signals by normalizing them to the *GAPDH* locus.

### Hi-C data analysis

The clean Hi-C reads were iteratively mapped to the human genome by the ICE software package^58^ (version 1f8815d0cc9e). QuASAR-Rep analysis (3DChromatinReplicateQC v 0.0.1)^59^ showed a high correlation between the two biological replicates, and we pooled the valid pairs of the two replicates together for further analysis. PCA of the normalized Hi-C matrices at 100-kb resolution was used for the identification of chromatin A/B compartments according to the published description^10^. The PC1 value of each bin defined the A (positive score) and B compartments (negative score). We determined the location and number of TADs and identified the locations of TAD borders using an insulation score algorithm^60^ based on normalized contact matrices at 40-kb resolution. The intra- and inter-chromosome interactions at 10-kb resolution were determined by Ay’s Fit-Hi-C software^20^ (v1.0.1). The interactions with *P*-value < 0.01, FDR < 0.01 and contact count > 2 were considered to be significant.

### ChIP-seq

ChIP-Seq assays were performed in duplicate as previously described^9^. Briefly, 1 × 10^7^ cells were fixed with 1% formaldehyde for 10 min. Cross-linked cells were lysed and sonicated (50 mM HEPES–NaOH, pH 7.5, 500 mM NaCl, 1 mM EDTA, 0.1% Na-deoxycholate, 1% TritonX-100, and 0.1% SDS) using a Covaris M220 to generate 300–500 bp DNA fragments. The Salt ion concentration of the cell lysate was then diluted to 300 mM for immunoprecipitation. Chromatin extract was incubated with primary antibodies overnight at 4 °C and then Dynabeads A/G beads (Invitrogen) were added to incubate for another 1 h. The beads with immunocomplexes were washed thrice in high-salt buffer, twice in low-salt buffer with lithium chloride, and once in TE buffer. After elution and de-crosslinking, DNA fragments were purified using the MinElute PCR Purification Kit (Qiagen). ChIP-Seq DNA libraries were constructed using KAPA Hyper Prep Kit (Kapa Biosystems, KK8502) and sequenced using the NovaSeq instrument. Primary antibodies used are as follows: p63 (CST, 13109, 10 μg/ChIP) CTCF (Millipore, 07-729, 10 μg/ChIP), SMC1 (Bethyl Laboratories, A300-055A, 10 μg/ChIP), EP300 (Abcam, ab14984, 10 μg/ChIP) and H3K9me2 (Abcam, ab1220, 10 μg/ChIP).

### ChIP-seq data analysis

ChIP-Seq data analysis was performed according to previous workflow^9^. Briefly, reads were trimmed using Trimmomatic tool^61^ and BWA software^62^ were used to align reads to human hg19 reference genome downloaded from Ensemble database. Picard Markduplicates tool was used to remove duplicated reads and only uniquely mapped reads were retained for downstream analysis. Peak callings for CTCF, SMC1, p63, and EP300 were achieved by MACS2^63^ with the parameters: -f BAMPE -B —SPMR -q 0.001 —call-summits —fix-bimodal —seed 11521 —extsize 200. For peak callings of H3K9me2 and H3K27me3, the following parameters were used: -f BAMPE -B —SPMR —fix-bimodal —extsize 500 —broad —broad-cutoff 0.01 —seed 11521 -c input file. HOMER mergePeaks tool was used to generate a list of merged peaks for two biological replicates. Bigwig files were generated by deepTools bamCoverage and visualized by Integrative Genomics Viewer. Chromatin states were annotated by ChromHMM (version 1.22)^64^. Motif analysis was performed using HOMER’s findMotifsGenome.pl program^65^. Intervene^66^ tool was used to intersect the SuReR-associated genes across distinct cell types.

### GO and KEGG enrichment analysis and identification of super-silencers

Super-silencers were identified and annotated to genes using the ROSE algorithm^29^. Clusterprofiler^67^ R package was used for enrichment analysis of GO biological processes with *p*-value cutoff = 0.01. The Metascape online tool^68^ was used for KEGG enrichment analysis.

### Statistics and graphing

The online imageGP tool (http://www.ehbio.com/Cloud_Platform/front/#/) was used to perform the ANOVA analysis and generate the violin plots, bubble plots of GO analysis, bar plots, and boxplots.

### Reporting summary

Further information on research design is available in the Nature Research Reporting Summary linked to this article.

## Supplementary information

Supplementary information

Reporting Summary

## Data Availability

The data that support this study are available from the corresponding authors upon reasonable request. Hi-C and ChIP-Seq data generated in the course of this study for CTCF, SMC1, p63, H3K9me2, and EP300 are available at the Gene Expression Omnibus (GEO) repository under the accession number GSE192625. Previously published data^7,9^ of RNA-Seq, ATAC-Seq, and ChIP-Seq for H3K27ac, H3K4me1, H3K4me3, H3K27me3, RUNX1, PAX6, and SMAD3 are available at GEO under the accession numbers GSE156273 and GSE155773. H3K27me3 ChIP-Seq data for IMR-90 (ENCSR431UUY) and myotube (ENCSR000ATI) were downloaded from Encyclopedia of DNA Elements (ENCODE). The previously published H3K27ac ChIP-seq data of head and neck squamous cell carcinoma and esophageal squamous cell carcinoma were obtained from GSE88976^69^ and GSE106433^70^. Source data are provided with this paper.

## Source data

Source Data

## Code availability

The code used for super-silencer identification is available at https://bitbucket.org/young_computation/rose^29^.
