# Supplementary material for: Comprehensive 3D epigenomic maps define limbal stem/progenitor cell function and identity
Source: Nat Commun. 2022 Mar 11;13:1293. doi: 10.1038/s41467-022-28966-6 (PMC8917218; doi:10.1038/s41467-022-28966-6)
Supplement: Supplementary file 1 — Supplementary information [file 41467_2022_28966_MOESM1_ESM.pdf]

Supplementary Information for

Comprehensive 3D epigenomic maps define limbal stem/progenitor cell function and identity

Mingsen Li[1], Huaxing Huang[1], Bofeng Wang[1], Shaoshuai Jiang[2], Huizhen Guo[1], Liqiong Zhu[1], Siqi Wu[1], Jiafeng Liu[1], Li Wang[1], Xihong Lan[1], Wang Zhang[1], Jin Zhu[1], Fuxi Li[1], Jieying Tan[1], Zhen Mao[1], Chunqiao Liu[1], Jianping Ji[1], Junjun Ding[2], Kang Zhang[3], Jin Yuan[1], Yizhi Liu[1,*] and Hong Ouyang [1,2,4,*]

*Correspondence: yzliu62@yahoo.com; Ouyhong3@mail.sysu.edu.cn

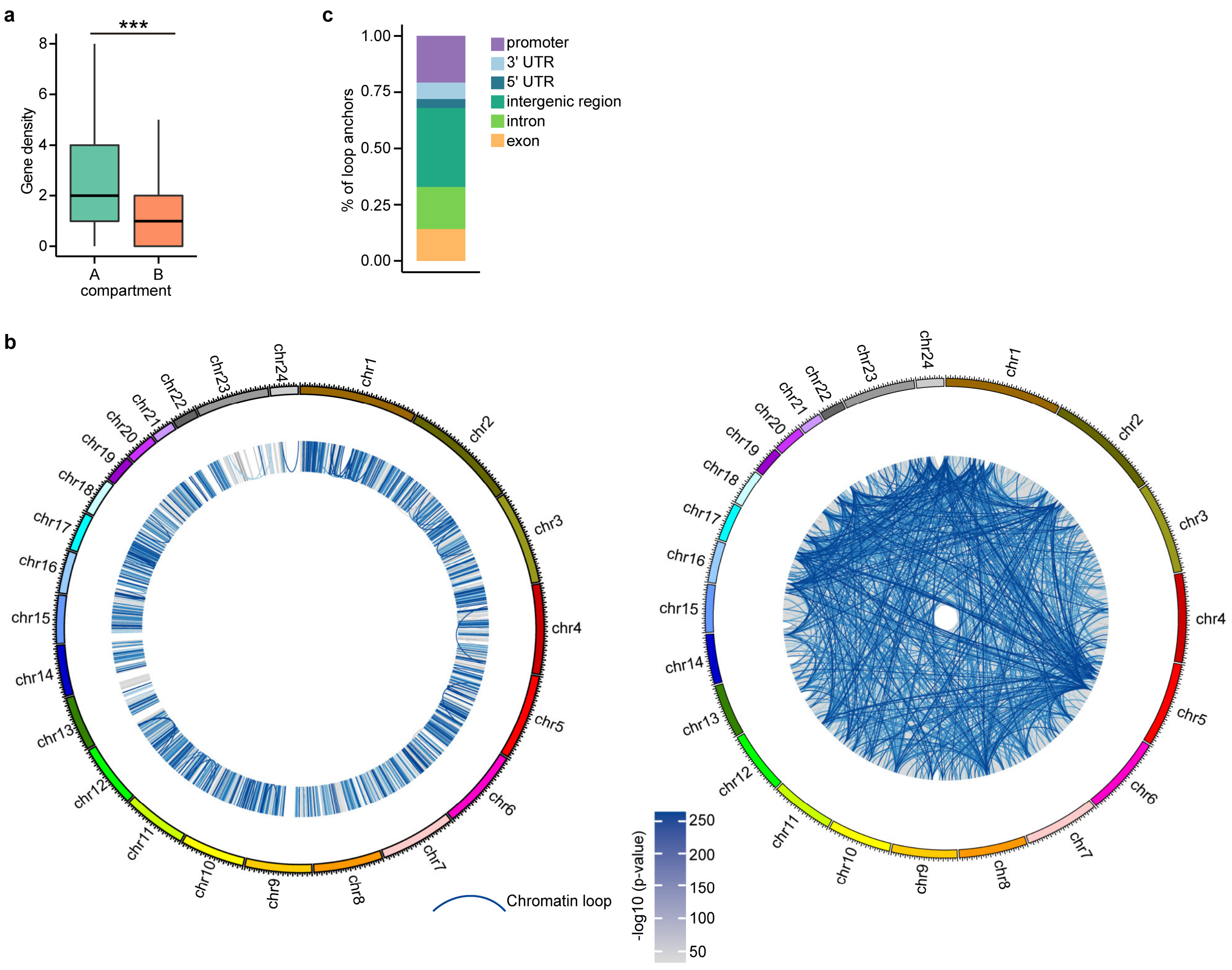

Supplementary Fig. 1 Characterization of Hi-C interactions in LSCs

a Boxplots showing gene density of A (n = 13,613) and B (n= 14,149) compartments. ****p* < 0.001 from two-way ANOVA. Boxplots indicate the 25th percentile (bottom of box), median (horizontal line inside box) and 75th percentile (top of box). Whiskers indicate 1.5 times the interquartile range. **b** Circos plots showing *cis*- and *trans*-interaction loops. **c** Distributions of chromatin loop anchors on the genome.

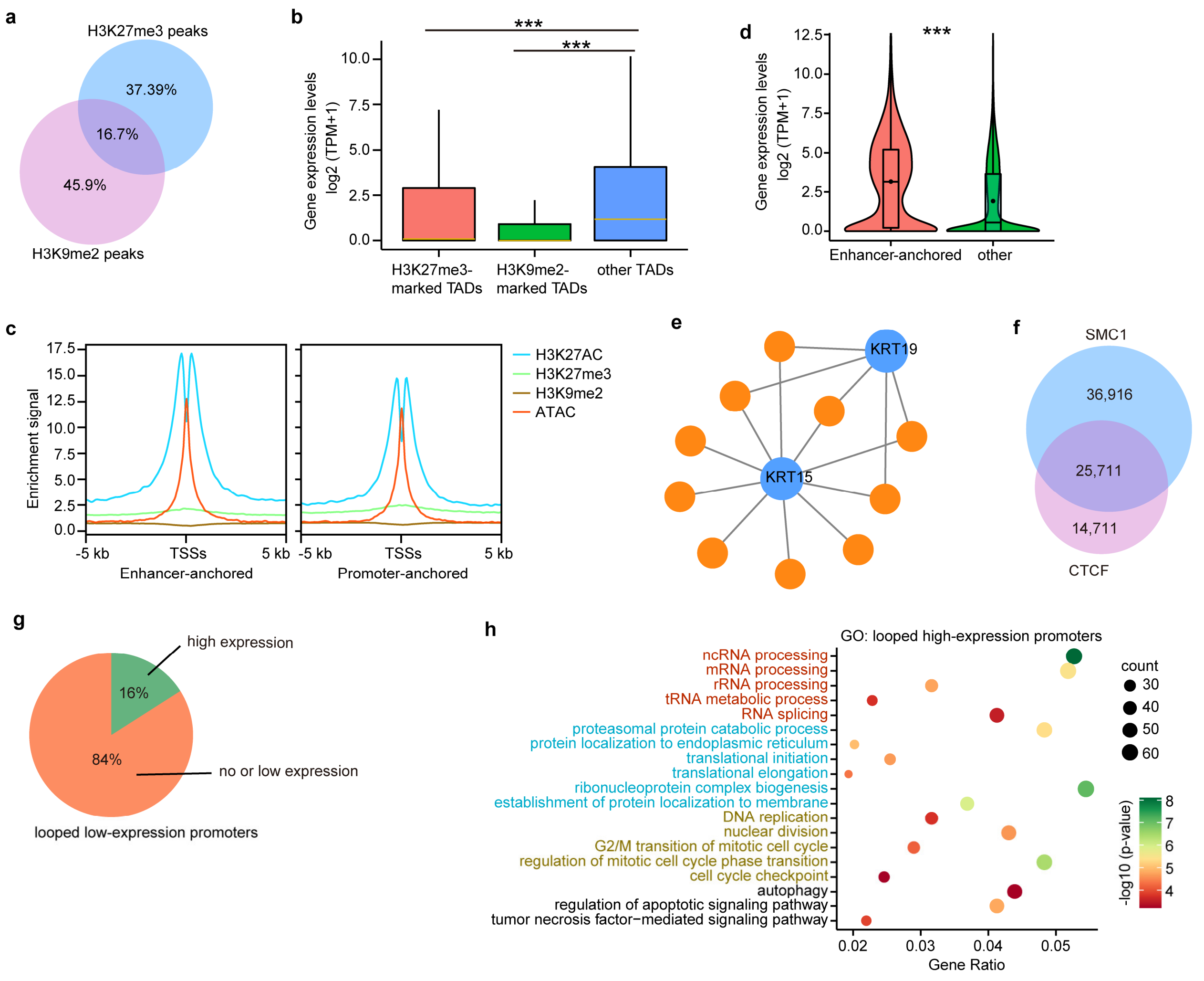

Supplementary Fig. 2 Epigenetic features of chromatin 3D structure in LSCs

a Venn diagram showing overlapping between H3K27me3 and H3K9me2 peaks. **b** Boxplots showing gene expression levels within H3K27me3-marked (n = 1,758), H3K9me2-marked (n = 495) and other (n = 23,050) TADs. ***$p <$ 0.001 from two-way ANOVA. Boxplots indicate the 25th percentile (bottom of box), median (horizontal yellow line inside box) and 75th percentile (top of box). Whiskers indicate 1.5 times the interquartile range. **c** Metaplots of ATAC-Seq and the indicated ChIP-Seq signals at enhancer-anchored and promoter-anchored TSSs. **d** Violin and box plots showing expression levels of enhancer-interacting (n=4,972) and other (n=20,107) genes. ***$p < 0.001$ from two-way ANOVA. Boxplots indicate the 25th percentile (bottom of box), median (horizontal line inside box), mean value (dark spot inside box) and 75th

percentile (top of box). Whiskers indicate 1.5 times the interquartile range. **e** A selected E-P interaction network. Orange nodes represent enhancers and blue nodes represent promoters of *KRT15* and *KRT19*. Edges represent loop interactions. **f** Overlapping between CTCF and SMC1 peaks. **g** Pie chart showing the distributions of expression levels of LLEP genes. **h** GO biological process analysis of the looped high-expression promoters that were identified in Fig .3h.

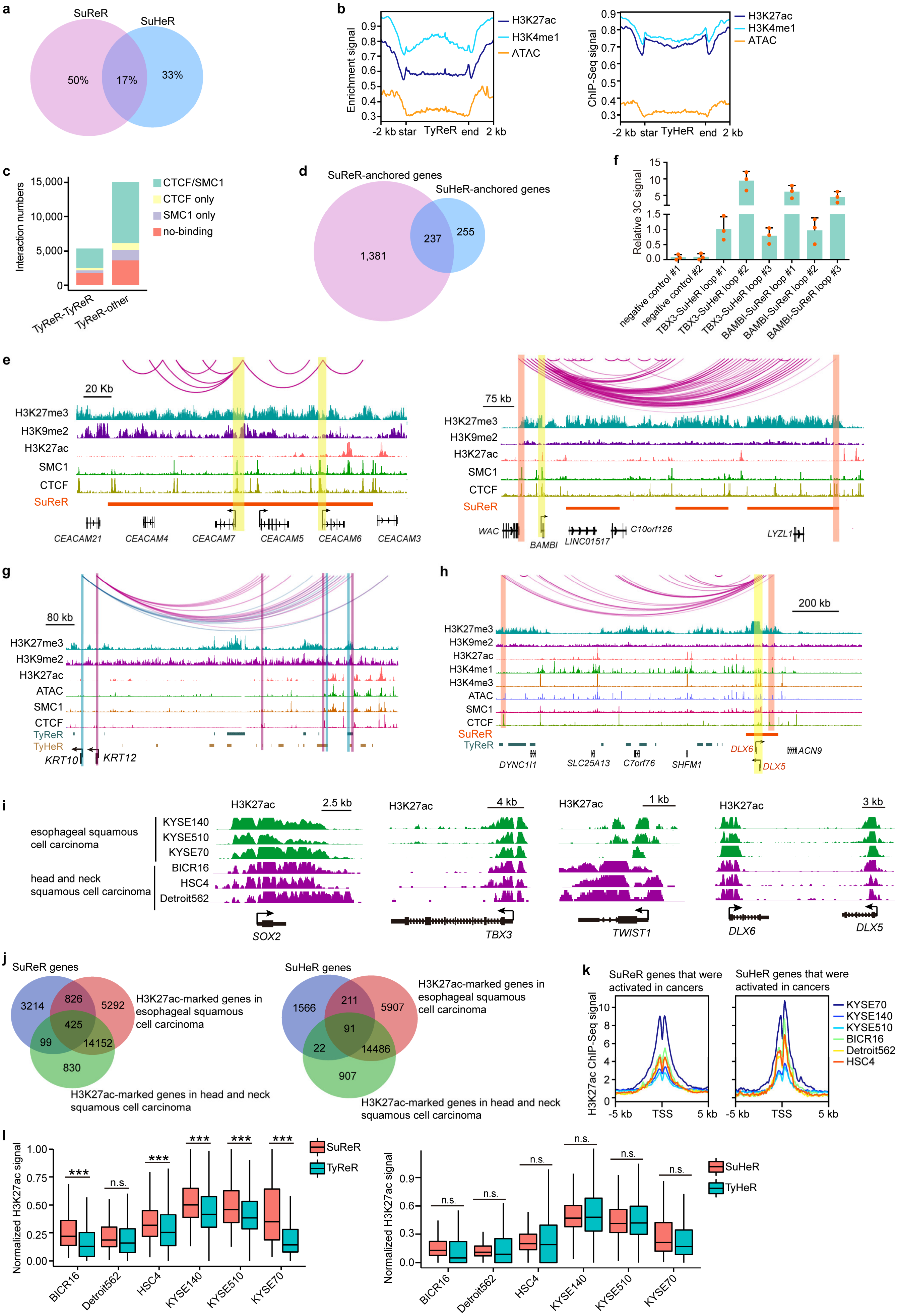

a
SuReR
SuHeR
50%
17%
33%
b
H3K27ac
H3K4me1
ATAC
Enrichment signal
-2 kb star TyReR end 2 kb
ChIP-Seq signal
-2 kb star TyHeR end 2 kb
c
Interaction numbers
CTCF/SMC1
CTCF only
SMC1 only
no-binding
TyReR-TyReR
TyReR-other
d
SuReR-anchored genes
SuHeR-anchored genes
1,381
237
255
f
Relative 3C signal
negative control #1
negative control #2
TBX3-SuHeR loop #1
TBX3-SuHeR loop #2
TBX3-SuHeR loop #3
BAMBI-SuReR loop #1
BAMBI-SuReR loop #2
BAMBI-SuReR loop #3
e
20 Kb
H3K27me3
H3K9me2
H3K27ac
SMC1
CTCF
SuReR
CEACAM21
CEACAM4
CEACAM7
CEACAM5
CEACAM6
CEACAM3
75 kb
WAC
BAMBI
LINC01517
C10orf126
LYZL1
g
80 kb
ATAC
TyReR
TyHeR
KRT10
KRT12
h
200 kb
H3K4me1
H3K4me3
DYNC1I1
SLC25A13
C7orf76
SHFM1
DLX6
DLX5
ACN9
i
esophageal squamous cell carcinoma
head and neck squamous cell carcinoma
KYSE140
KYSE510
KYSE70
BICR16
HSC4
Detroit562
2.5 kb
4 kb
1 kb
3 kb
SOX2
TBX3
TWIST1
j
SuReR genes
H3K27ac-marked genes in esophageal squamous cell carcinoma
H3K27ac-marked genes in head and neck squamous cell carcinoma
3214
826
5292
425
99
14152
830
SuHeR genes
1566
211
5907
91
22
14486
907
k
SuReR genes that were activated in cancers
SuHeR genes that were activated in cancers
H3K27ac ChIP-Seq signal
-5 kb
TSS
5 kb
l
Normalized H3K27ac signal
SuReR
TyReR
SuHeR
TyHeR
***
n.s.

Supplementary Fig. 3 Characterization of super-silencer-associated interactions

a Overlapping between SuReR and SuHeR. **b** Metaplots showing the enrichment of H3K27ac, H3K4me1 and ATAC at TyReR and TyHeR. **c** Distributions of TyReR-TyReR and TyReR-other loops that were occupied by CTCF only, SMC1 only, CTCF/SMC1 and were not bound by CTCF and SMC1. **d** Overlapping between SuReR-anchored and SuHeR-anchored genes. **e** Identified chromatin loops and tracks for the indicated ChIP-Seq enrichment at the indicated genome loci. **f** Chromosome conformation capture (3C) assay for TBX3-SuHeR and BAMBI-SuReR loops that were identified in Hi-C data. Values are shown as means ± SD (n = 3 biologically independent experiments). Source data are provided as a Source Data file. **g**, **h** Identified chromatin loops and tracks for the indicated ChIP-Seq enrichment at the indicated genome loci. **i** Tracks for H3K27ac ChIP-Seq enrichment at the indicated genome loci in head and neck squamous cell carcinoma (BICR16, HSC4 and Detroit562) and esophageal squamous cell carcinoma (KYSE140, KYSE510 and KYSE70) cell lines. **j** Overlap among super-silencer genes, H3K27ac-marked genes in head and neck squamous cell carcinoma and H3K27ac-marked genes in esophageal squamous cell carcinoma. **k** Metaplots of H3K27ac ChIP-Seq signals of the indicated squamous cell carcinoma cell lines at the TSSs of the overlapped genes in **j**. **l** The normalized H3K27ac ChIP-Seq enrichment signal of the indicated cell lines at SuReR (n=1,130), TyReR (n=15,653), SuHeR (n=896) and TyHeR (n=33,691) identified in LSCs. ***$p < 0.001$ from two-way ANOVA. n.s. indicates not significant. Boxplots indicate the 25th percentile (bottom of box), median (horizontal line inside box) and 75th percentile (top of box). Whiskers indicate 1.5 times the interquartile range.

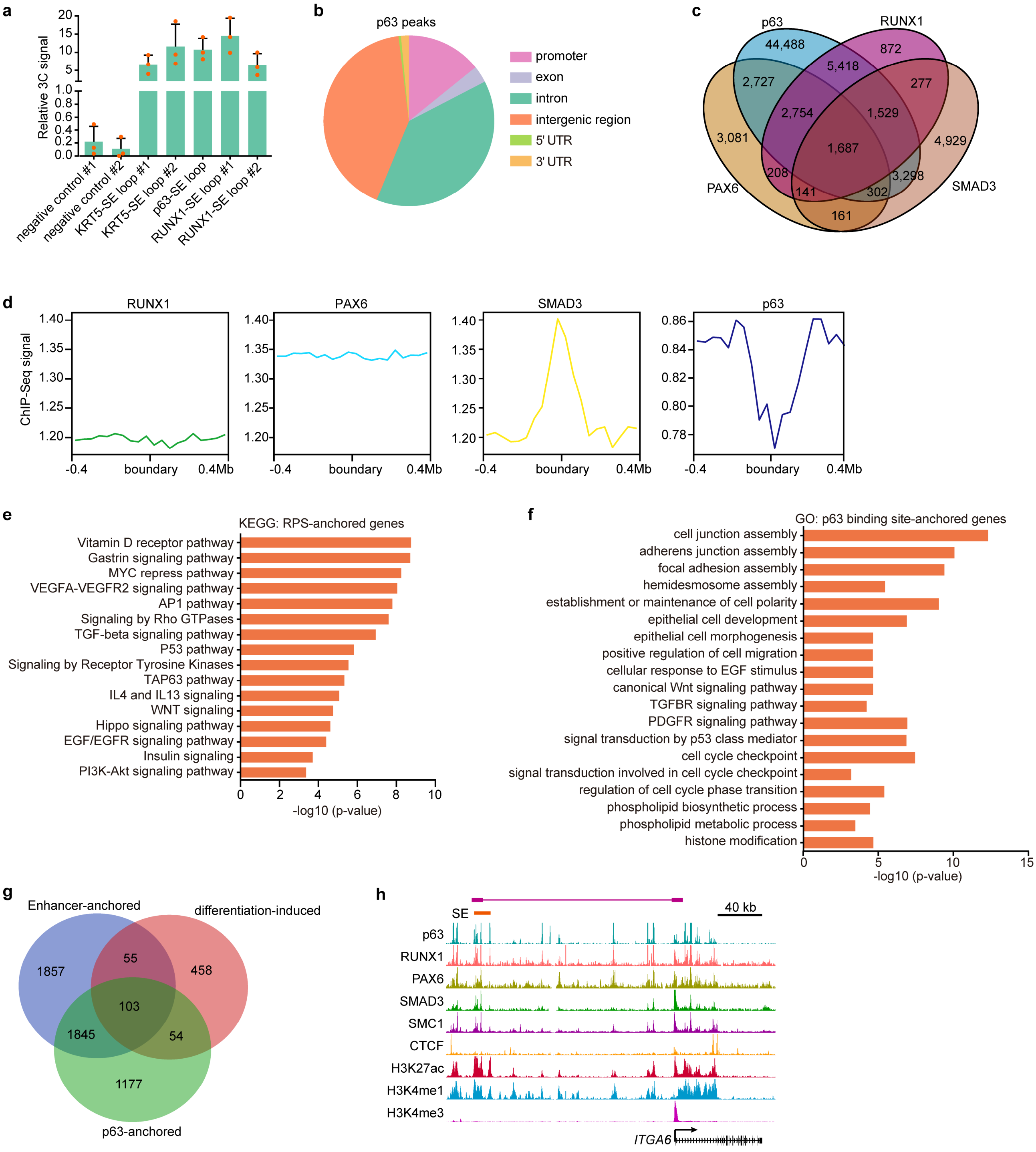

Supplementary Fig. 4 Binding pattern of p63 and RPS and their associated chromatin interactions

a 3C assay for KRT5-SE, p63-SE and RUNX1-SE loops that were identified in Hi-C data. Values are shown as means ± SD (n = 3 biologically independent experiments). Source data are provided as a Source Data file. **b** Pie chart showing the distributions of p63 peaks on the genome. **c** Overlapping among RPS and p63 peaks. **d** Metaplots showing the enrichment of RPS and p63 at

the TAD boundaries. **e** Kyoto Encyclopedia of Genes and Genomes pathway analysis for the RPS-anchored genes. **f** GO analysis for genes that interacted with p63 binding sites. **g** Vene showing the overlap among differentiation-induced, enhancer-anchored and p63-anchored genes. **h** A identified chromatin loop and tracks for the indicated ChIP-Seq signals at *ITGA6* locus.
