# Supplementary material for: Comprehensive 3D epigenomic maps define limbal stem/progenitor cell function and identity
Source: Nat Commun. 2022 Mar 11;13:1293. doi: 10.1038/s41467-022-28966-6 (PMC8917218; doi:10.1038/s41467-022-28966-6)
Supplement: Supplementary file 2 — Reporting Summary [file 41467_2022_28966_MOESM2_ESM.pdf]

nature portfolio

Corresponding author(s): Hong Ouyang

Last updated by author(s): Jan 28, 2022

Reporting Summary

Nature Portfolio wishes to improve the reproducibility of the work that we publish. This form provides structure for consistency and transparency in reporting. For further information on Nature Portfolio policies, see our Editorial Policies and the Editorial Policy Checklist.

Statistics

For all statistical analyses, confirm that the following items are present in the figure legend, table legend, main text, or Methods section.

n/a	Confirmed	
☐	☒	The exact sample size (*n*) for each experimental group/condition, given as a discrete number and unit of measurement
☐	☒	A statement on whether measurements were taken from distinct samples or whether the same sample was measured repeatedly
☐	☒	The statistical test(s) used AND whether they are one- or two-sided<br>*Only common tests should be described solely by name; describe more complex techniques in the Methods section.*
☒	☐	A description of all covariates tested
☐	☒	A description of any assumptions or corrections, such as tests of normality and adjustment for multiple comparisons
☐	☒	A full description of the statistical parameters including central tendency (e.g. means) or other basic estimates (e.g. regression coefficient) AND variation (e.g. standard deviation) or associated estimates of uncertainty (e.g. confidence intervals)
☐	☒	For null hypothesis testing, the test statistic (e.g. F, t, r) with confidence intervals, effect sizes, degrees of freedom and P value noted<br>*Give P values as exact values whenever suitable.*
☒	☐	For Bayesian analysis, information on the choice of priors and Markov chain Monte Carlo settings
☐	☒	For hierarchical and complex designs, identification of the appropriate level for tests and full reporting of outcomes
☐	☒	Estimates of effect sizes (e.g. Cohen's *d*, Pearson's *r*), indicating how they were calculated

Our web collection on statistics for biologists contains articles on many of the points above.

Software and code

Policy information about availability of computer code

Data collection	Sequencing data was obtained from the HiSeq X Ten and NovaSeq 6000 systems (Illumina).
Data analysis	trimmomatic tool (version 0.36), BWA (version 0.7.17), MACS2 (version 2.1.1), HOMER (v4.9.1), deepTools (v3.0.2), clusterprofiler (v3.8.1), ICE software package (version 1f8815d0cc9e), 3DChromatinReplicateQC (v 0.0.1), Ay's Fit-Hi-C software (v1.0.1), ChromHMM (version 1.22), Intervene (version 0.5.8), Metascape (version 1.0), Picard Markduplicates (version 2.18.16), Integrative Genomics Viewer (version 2.4.13) and ROSE (version 1.0).

For manuscripts utilizing custom algorithms or software that are central to the research but not yet described in published literature, software must be made available to editors and reviewers. We strongly encourage code deposition in a community repository (e.g. GitHub). See the Nature Portfolio guidelines for submitting code & software for further information.

Data

Policy information about availability of data

All manuscripts must include a data availability statement. This statement should provide the following information, where applicable:

- Accession codes, unique identifiers, or web links for publicly available datasets
- A description of any restrictions on data availability
- For clinical datasets or third party data, please ensure that the statement adheres to our policy

All data supporting the findings of this study are available within the paper and its supplementary information file. Previously published data of RNA-Seq, ATAC-Seq and ChIP-Seq for H3K27ac, H3K4me1, H3K4me3, H3K27me3, RUNX1, PAX6 and SMAD3 are available at the Gene Expression Omnibus repository under the accession number GSE156273 and GSE155773. H3K27me3 ChIP-Seq data for IMR-90 (ENCSR431UUY) and myotube (ENCSR000ATI) were downloaded from Encyclopedia of DNA Elements. The previously published H3K27ac ChIP-seq data of head and neck squamous cell carcinoma and esophageal squamous cell

carcinoma were obtained from GSE8897669 and GSE10643370. Hi-C and ChIP-Seq data for CTCF, SMC1, p63, H3K9me2 and EP300 are available at the Gene Expression Omnibus repository under the accession number GSE192625.

Field-specific reporting

Please select the one below that is the best fit for your research. If you are not sure, read the appropriate sections before making your selection.

- [x] Life sciences
- [ ] Behavioural & social sciences
- [ ] Ecological, evolutionary & environmental sciences

For a reference copy of the document with all sections, see nature.com/documents/nr-reporting-summary-flat.pdf

Life sciences study design

All studies must disclose on these points even when the disclosure is negative.

Sample size	Chromosome Conformation Capture (3C) experiment was performed in triplicate and the next-generation sequencing experiments were performed in two replicates, which is sufficient to determine reproducible results based on extensive experience and is widely accepted. No statistical methods were used to determine sample sizes. All the sample sizes of sequencing data were based on standard sequencing analysis practices and were determined to be suitable for statistical analyses.
Data exclusions	No data were excluded.
Replication	Hi-C and ChIP-seq experiments were performed for 2 biological replicates and 3C experiment was conducted for three biological replicates.
Randomization	This study does not involve work that requires random allocation because all experiments are performed in one cell type and no comparisons between experimental groups were made.
Blinding	Blinding was not relevant to this study, because all experiments were conducted on cell cultures without human or animal subjectivity

Reporting for specific materials, systems and methods

We require information from authors about some types of materials, experimental systems and methods used in many studies. Here, indicate whether each material, system or method listed is relevant to your study. If you are not sure if a list item applies to your research, read the appropriate section before selecting a response.

Materials & experimental systems

n/a	Involved in the study	
☐	☒	Antibodies
☒	☐	Eukaryotic cell lines
☒	☐	Palaeontology and archaeology
☒	☐	Animals and other organisms
☐	☒	Human research participants
☒	☐	Clinical data
☒	☐	Dual use research of concern

Methods

n/a	Involved in the study	
☐	☒	ChIP-seq
☒	☐	Flow cytometry
☒	☐	MRI-based neuroimaging

Antibodies

Antibodies used	Antibodies used for ChIP-seq (10μg/ChIP): p63 (CST, 13109), CTCF (Millipore, 07-729), SMC1 (Bethyl Laboratories, A300-055A), EP300 (Abcam, ab14984) and H3K9me2 (Abcam, ab1220)
Validation	Antibodies used in our study were validated as noted by suppliers. Antibody validation information and relevant citations can be found on manufacturers' website.<br>p63: https://www.cellsignal.cn/products/primary-antibodies/p63-a-d2k8x-xp-rabbit-mab/13109?site-search-type=Products&N=4294956287&Ntt=13109&fromPage=plp&_requestid=752781<br>CTCF: https://www.sigmaaldrich.cn/CN/zh/product/mm/07729<br>SMC1: https://www.biomol.com/products/antibodies/primary-antibodies/general/anti-smc1-a300-055a-t?number=A300-055A<br>EP300: https://www.abcam.cn/kat3b-p300-antibody-3g230-nm-11-chip-grade-ab14984.html<br>H3K9me2: https://www.abcam.cn/histone-h3-di-methyl-k9-antibody-mabcam-1220-chip-grade-ab1220.html

Human research participants

Policy information about studies involving human research participants

Population characteristics	All human limbus tissues were obtained from four male donors aged from 20 to 46. These donors do not have any ocular surface diseases.
Recruitment	This study did not involve recruitment of patients. Normal human limbus of donors were obtained from eye bank of Zhongshan Ophthalmic Center.
Ethics oversight	All human tissues were obtained with the approval of the Ethics Committee of Zhongshan Ophthalmic Center of Sun Yat-sen University.

Note that full information on the approval of the study protocol must also be provided in the manuscript.

ChIP-seq

Data deposition

- [x] Confirm that both raw and final processed data have been deposited in a public database such as GEO.
- [x] Confirm that you have deposited or provided access to graph files (e.g. BED files) for the called peaks.

Data access links<br>*May remain private before publication.*	https://www.ncbi.nlm.nih.gov/geo/query/acc.cgi?acc=GSE156273<br>https://www.ncbi.nlm.nih.gov/geo/query/acc.cgi?acc=GSE88976<br>https://www.ncbi.nlm.nih.gov/geo/query/acc.cgi?acc=GSE106433<br>https://www.ncbi.nlm.nih.gov/geo/query/acc.cgi?acc=GSE192625
Files in database submission	H3K27ac_rep1<br>H3K27ac_rep2<br>H3K4me1_rep1<br>H3K4me1_rep2<br>H3K4me3_rep1<br>H3K4me3_rep2<br>H3K27me3_rep1<br>H3K27me3_rep2<br>RUNX1_rep1<br>RUNX1_rep2<br>PAX6_rep1<br>PAX6_rep2<br>SMAD3_rep1<br>SMAD3_rep2<br>H3K9me2_rep1<br>H3K9me2_rep2<br>CTCF_rep1<br>CTCF_rep2<br>SMC1_rep1<br>SMC1_rep2<br>EP300_rep1<br>EP300_rep2<br>p63_rep1<br>p63_rep2<br>H3K27ac-BICR16<br>H3K27ac-HSC4<br>H3K27ac-Detroit562<br>KYSE140_H3K27Ac<br>KYSE510_H3K27Ac<br>KYSE70_H3K27Ac
Genome browser session<br>(e.g. UCSC)	GSE156273, GSE88976, GSE106433 and GSE192625

Methodology

Replicates	Two independent experimental replications were performed.
Sequencing depth	6Gb paired-end data were generated.
Antibodies	Antibodies used in this work: p63 (CST, 13109), CTCF (Millipore, 07-729), SMC1 (Bethyl Laboratories, A300-055A), EP300 (Abcam, ab14984) and H3K9me2 (Abcam, ab1220).

Peak calling parameters	-f BAMPE -B --SPMR -q 0.001 --call-summits --fix-bimodal --seed 11521 --extsize 200
Data quality	FastQC(v0.11.8) was used to assess the data qualit
Software	trimmomatic tool (version 0.36), BWA (version 0.7.17), MACS2 (version 2.1.1), deepTools (v3.0.2), HOMER (v4.9.1),
